# Disruption to *de novo* uridine biosynthesis alters β-1,3-glucan masking in *Candida albicans*

**DOI:** 10.1128/msphere.00287-24

**Published:** 2024-08-08

**Authors:** Mikayla M. Mangrum, Amanda K. Vogel, Andrew S. Wagner, Ainsley E. King, Jian Miao, Yue Zhou, Elise K. Phillips, Brian M. Peters, Todd B. Reynolds

**Affiliations:** 1 Department of Microbiology, University of Tennessee, Knoxville, Tennessee, USA; 2 Integrated Program in Biomedical Sciences, College of Graduate Health Sciences, University of Tennessee Health Science Center, Memphis, Tennessee, USA; 3 Pharmaceutical Sciences Program, College of Graduate Health Sciences, University of Tennessee Health Science Center, Memphis, Tennessee, USA; 4 Department of Clinical Pharmacy and Translational Science, College of Pharmacy, University of Tennessee Health Science Center, Memphis, Tennessee, USA; 5 Department of Microbiology, Immunology, and Biochemistry, College of Medicine, University of Tennessee Health Science Center, Memphis, Tennessee, USA; University of Guelph, Guelph, Ontario, Canada

**Keywords:** *Candida*, *ura3*, glucan, cell wall, virulence, unmasking

## Abstract

The uridine derivatives UDP-glucose and UDP-*N*-acetylglucosamine are important for cell wall construction as they are the precursors for the synthesis of β-1,3-glucan and chitin, respectively. Previous studies have demonstrated attenuated virulence of uridine auxotrophs in mice, which has been attributed to insufficient uridine levels for growth in the host. We have discovered that uridine deprivation in the uridine auxotroph *ura3*ΔΔ disrupts cell wall architecture by increasing surface mannans, exposing β-1,3-glucan and chitin, and decreasing UDP-sugar levels. Cell wall architecture and UDP-sugars can be rescued with uridine supplementation. The cell wall architectural disruptions in the *ura3*ΔΔ mutant also impact immune activation since the mutant elicited greater TNFα secretion from RAW264.7 macrophages than wild type. To determine if cell wall defects contributed to decreased virulence in the *ura3*ΔΔ mutant, we used a murine model of systemic infection. Mice infected with the *ura3*ΔΔ mutant exhibited increased survival and reduced kidney fungal burden compared with mice infected with wild type. However, suppression of the immune response with cyclophosphamide did not rescue virulence in mice infected with the *ura3*ΔΔ mutant, indicating the attenuation in virulence of uridine auxotrophs can be attributed to decreased growth in the host but not increased exposure of β-1,3-glucan. Moreover, the *ura3*ΔΔ mutant is unable to grow on *ex vivo* kidney agar, which demonstrates its inability to colonize the kidneys due to poor growth. Thus, although uridine auxotrophy elicits changes to cell wall architecture that increase the exposure of immunogenic polymers, metabolic fitness costs more strongly drive the observed virulence attenuation.

**IMPORTANCE:**

*Candida albicans* is a common cause of bloodstream infections (candidemia). Treatment of these bloodstream infections is made difficult because of increasing antifungal resistance and drug toxicity. Thus, new tactics are needed for antifungal drug development, with immunotherapy being of particular interest. The cell wall of *C. albicans* is composed of highly immunogenic polymers, particularly β-1,3-glucan. However, β-1,3-glucan is naturally masked by an outer layer of mannoproteins, which hampers the detection of the fungus by the host immune system. Alteration in cell wall components has been shown to increase β-1,3-glucan exposure; however, it is unknown how the inability to synthesize precursors to cell wall components affects unmasking. Here, we demonstrate how cell wall architecture is altered in response to a deficit in precursors for cell wall synthesis and how uridine is a crucial component of these precursors.

## INTRODUCTION

*Candida albicans* is an opportunistic fungal pathogen that colonizes various host mucosal surfaces such as those in the oropharyngeal, vulvovaginal, and gastrointestinal tracts (1–3). In more serious cases, *Candida* may enter the bloodstream and disseminate to organs causing systemic candidiasis (4, 5). *Candida* species are the fourth leading cause of nosocomial bloodstream infections, exhibiting rising antifungal resistance, and there are increased infection rates with non-*albicans Candida* species (6–10). This underscores the importance of understanding *Candida* spp. pathogenicity and colonization of various host niches. To be a successful pathogen, *C. albicans* must overcome stressors imposed by the host, especially in the maintenance of its nutritional requirements against host sequestering of nutrients (11–13).

*C. albicans* may acquire nutrients such as amino acids, lipids, nucleosides, and other metabolites through *de novo* biosynthetic and salvage pathways. *C. albicans* possesses amino acid permeases and proteinases for the uptake of amino acids and contains biosynthetic pathways for essential amino acids (14–16). Although the *de novo* pathways for the biosynthesis of amino acids such as histidine, leucine, and arginine are dispensable for virulence (likely due to sufficient availability in the host), disruption of the *de novo* biosynthesis of other metabolites alters *C. albicans* virulence (17). Glutathione auxotrophy attenuates virulence in a murine systemic infection, and heme biosynthesis is required for full virulence *in vivo* (18, 19). Furthermore, the *de novo* biosynthesis of lipids is critical for virulence. Disruption of *de novo* phospholipid biosynthesis by the deletion of *CHO1* (encoding phosphatidylserine synthase) results in an ethanolamine auxotroph with decreased virulence *in vivo* (20, 21). Additionally, a *fas2*ΔΔ mutant, defective in fatty-acid synthase activity, is avirulent in a mouse model of systemic infection and a rat model of oropharyngeal infection (22, 23). Thus, the ability to utilize biosynthetic pathways for specific nutrients is crucial for growth and survival during infection.

Nucleosides are important for a variety of cellular processes through their direct roles as subunits of nucleic acids as well as additional roles as precursors in essential polymers. Metabolism of nucleosides has been characterized in *Saccharomyces cerevisiae* and is likely relatively conserved throughout yeast species (24). The *de novo* pyrimidine biosynthetic pathway in yeast consists of a multistep process in which precursors (L-glutamine and bicarbonate, L-aspartate, or phosphoribosyl pyrophosphate) are converted to uridine monophosphate (UMP) (25, 26). Deletion of one key gene in this pathway, *URA3*, results in a uridine auxotroph, which has been well studied due to its use as a selectable marker for the genetic manipulation of fungi (27–29). *URA3* encodes the enzyme orotidine 5′-monophosphate decarboxylase, which converts orotidine 5′-monophosphate to UMP that may be further converted to make uridine diphosphate (UDP), uridine triphosphate (UTP), or cytidine triphosphate (CTP) (26). The pyrimidine salvage pathway works in parallel to the *de novo* pathway and converges at the point of UMP production (30). Exogenous uridine or cytidine is converted to uracil, which is subsequently converted to UMP (31). Phosphorylated forms of uridine can be used in the synthesis of RNA or in other metabolic processes including cell wall synthesis.

The cell wall of *C. albicans* is composed of highly immunogenic β-1,3-glucan, as well as β-1,6-glucan and chitin (32). However, these polysaccharides are covered, or “masked,” by an outer layer of mannoproteins, which impair immune detection and hamper fungal clearance (33–37). Increasing the exposure of β-1,3-glucan decreases fungal burden and virulence *in vivo* and increases immune activation *in vitro* (20, 36, 38). Exposure to these polysaccharides is modulated by a variety of environmental stressors including echinocandin treatment and host immune responses (35, 39, 40). The precursors to β-glucans and chitin are UDP-glucose and UDP-*N*-acetylglucosamine, respectively (41, 42). Thus, cell wall architecture depends on the availability of uridine, which forms some of the precursors for cell wall polysaccharides.

Uridine auxotrophic strains are adhesion-deficient and significantly attenuated in virulence in murine systemic infections (19, 43, 44). This has been attributed to a lack of intracellular uridine decreasing fitness in the host, presumably based on decreased transcription and growth. Additionally, genome position-dependent effects of *URA3* in virulence and physiology have also been observed and reviewed extensively (45, 46). In this study, we show that a uridine auxotroph also has a disorganized cell wall that is rescued with uridine supplementation. Interestingly, although total levels of chitin and β-glucans do not decrease in a uridine auxotroph, the levels of UDP-GlcNAc and UDP-glucose are reduced. This coincides with an increase in β-1,3-glucan synthase and chitin synthase expression. Although this may contribute to host recognition, we observed that the attenuated virulence of a uridine auxotroph is not host immune system dependent, indicating that a growth defect is the primary driver of reduced virulence in the *ura3*ΔΔ mutant.

## RESULTS

### Uridine auxotrophy increases β-1,3-glucan exposure and decreases growth

**Fig 1:**
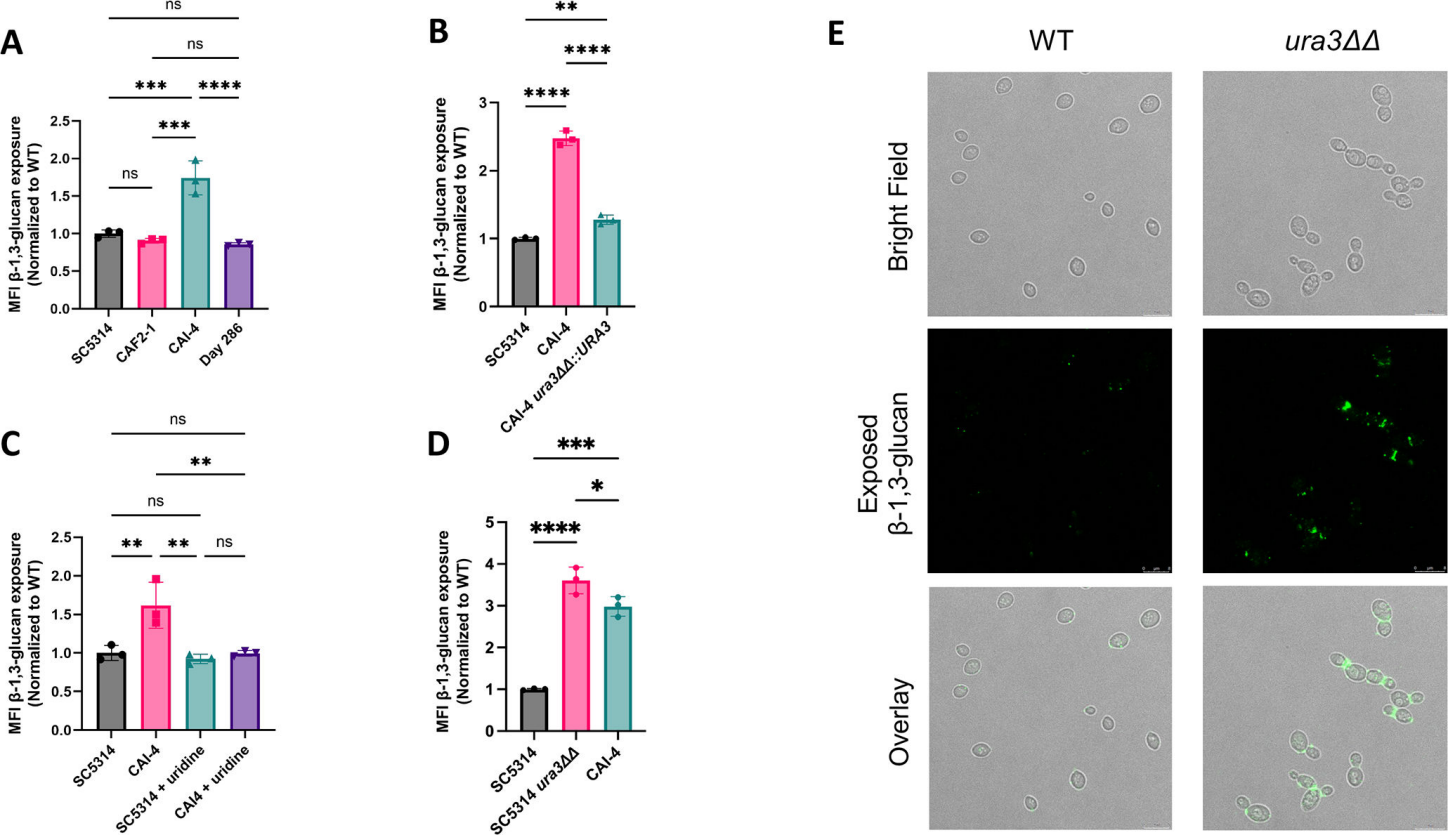
Uridine auxotrophy slows growth and increases β-1,3-glucan exposure. (**A–E**) Strains were grown overnight in YPD and then stained with an anti-β-1,3-glucan antibody and a phycoerythrin-conjugated secondary antibody for flow cytometry or confocal microscopy, and the MFI of β-1,3-glucan exposure was measured. (**A**) CAI-4 was compared with SC5314 and other lab strains. (*n* = 3 biological replicates with one technical replicate for each, ***P < 0.0005, ****P < 0.0001, by one-way analysis of variance (ANOVA)). (**B**) *URA3* was complemented into CAI-4 and measured for β-1,3-glucan masking. (*n* = 3 biological replicates with one technical replicate for each, **P < 0.01, ****P < 0.0001, by one-way ANOVA.) (**C**) SC5314 and CAI-4 were grown overnight in YPD with or without 100 µg/mL uridine supplementation, and β-1,3-glucan exposure was assessed. (*n* = 3 biological replicates with one technical replicate for each, **P < 0.01, by one-way ANOVA.) (**D**) SC5314 *ura3*ΔΔ was measured for β-1,3-glucan exposure in comparison to CAI-4. (*n* = 3 biological replicates with one technical replicate for each, *P < 0.05, ***P < 0.0005, ****P < 0.0001, by one-way ANOVA.) (**E**) Confocal microscopy images of SC5314 and SC5314 *ura3*ΔΔ stained for exposed β-1,3-glucan (scale bar indicates 10 µm).

*C. albicans* CAI-4 is a uridine auxotroph as it does not contain any functional copies of the gene *URA3*, whereas the parental strain and other derivatives (e.g., CAF2-1, DAY286) carry at least one functional copy (28). To assess the impact that *URA3* copy number has on unmasking, we compared the β-1,3-glucan exposure between these strains by immunofluorescent staining for flow cytometry. Surprisingly, CAI-4 (*ura3*ΔΔ) exhibited significantly higher unmasking of β-1,3-glucan than all other strains tested (Fig. 1A). Complementation of CAI-4 with *URA3* significantly reduced β-1,3-glucan exposure toward the level observed in the parental wild-type strain SC5314 (Fig. 1B). Additionally, complementation of *URA3* into CAI-4 rescues the growth defect of CAI-4 in yeast peptone dextrose (YPD) (Fig. S1). Since the pyrimidine salvage pathway is still functional in a uridine auxotroph, we investigated whether supplementation of uridine into the growth media (YPD) would also reduce the level of β-1,3-glucan exposure. We observed that growth in YPD with 100 µg/mL uridine decreased β-1,3-glucan exposure in CAI-4 (Fig. 1C). Although originally derived from SC5314, CAI-4 contains several genetic differences including alterations in ploidy (28). To determine if the parental SC5314 strain would also exhibit β-1,3-glucan unmasking when *URA3* is disrupted, we deleted *URA3* in the SC5314 background and found that this similarly increased β-1,3-glucan exposure (Fig. 1D). Confocal microscopy of the SC5314 *ura3*ΔΔ mutant showed more foci of β-1,3-glucan unmasking along a greater portion of the cell’s periphery than wild type (Fig. 1E). Thus, uridine, acquired either through *de novo* biosynthesis or pyrimidine salvage, is required for β-1,3-glucan masking. Given that SC5314 is a commonly used lab-domesticated strain, we utilized the *ura3*ΔΔ strain created in this background for the remainder of experiments in this study.

### Exposure of cell wall components in uridine auxotrophs is reduced by exogenous uridine

**Fig 2:**
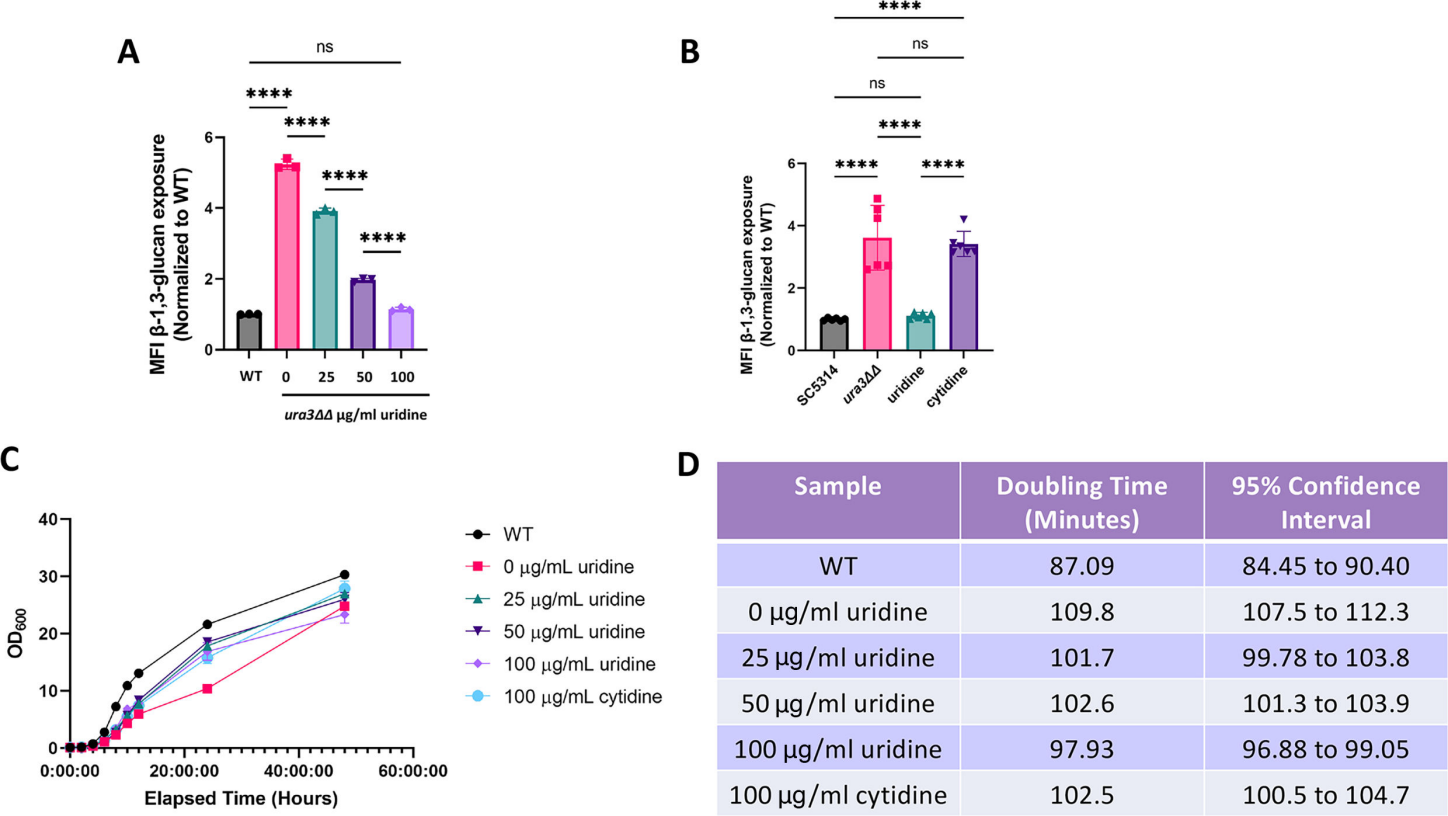
Supplementation of the *ura3*ΔΔ mutant with exogenous uridine restores growth and β-1,3-glucan masking. (**A**) Wild-type and *ura3*ΔΔ cells were grown overnight in YPD with 0, 25, 50, or 100 µg/mL uridine and stained with an anti-β-1,3-glucan antibody and a phycoerythrin-conjugated secondary antibody for flow cytometry, and the MFI of exposed β-1,3-glucan was measured. (*n* = 3 biological replicates with one technical replicate for each, ****P < 0.0001, by one-way ANOVA.) (**B**) Cells were grown overnight in YPD or YPD with 100 µg/mL uridine or cytidine and measured for exposed β-1,3-glucan. (*n* = 6 biological replicates with one technical replicate for each, ****P < 0.0001, by one-way ANOVA.) (**C**) Cells were grown overnight and then back diluted and measured for optical density (OD_600_) every 2 hours for 12 hours and at the 24- and 48-hour time points. Error bars represent the standard deviation. (**D**) Doubling time was calculated by Malthusian exponential growth from the 4- to 10-hour time points. The 95% CI (profile likelihood) is also shown (*n* = 3 biological replicates).

The observation that supplementation with 100 µg/mL uridine reduces unmasking in the uridine auxotroph raises the possibility of a dose-dependent effect of uridine concentration on cell wall architecture. We hypothesized that β-1,3-glucan exposure would decrease, as uridine concentration in the media increased due to the higher availability of uridine for salvage. To test this, we measured β-1,3-glucan exposure in SC5314 *ura3*ΔΔ cells grown overnight in YPD with 0, 25, 50, or 100 µg/mL uridine. An inverse correlation between unmasking and uridine concentration was observed (Fig. 2A). In the pyrimidine salvage pathway, exogenous cytidine can be acquired and used by the cell directly or can be converted to uridine (30). We hypothesized that the addition of cytidine into the growth media would similarly decrease β-1,3-glucan exposure and restore the growth of the uridine auxotroph due to cytidine-to-uridine conversion. However, supplementation of YPD with 100 µg/mL cytidine did not decrease β-1,3-glucan exposure (Fig. 2B). Furthermore, supplementation of other nucleosides (adenosine and guanosine) into the growth media did not decrease unmasking (Fig. S2). Thus, supplementation with nucleosides beside uridine is unable to decrease β-1,3-glucan exposure of the *ura3*ΔΔ strain to wild-type levels.

**Fig 3:**
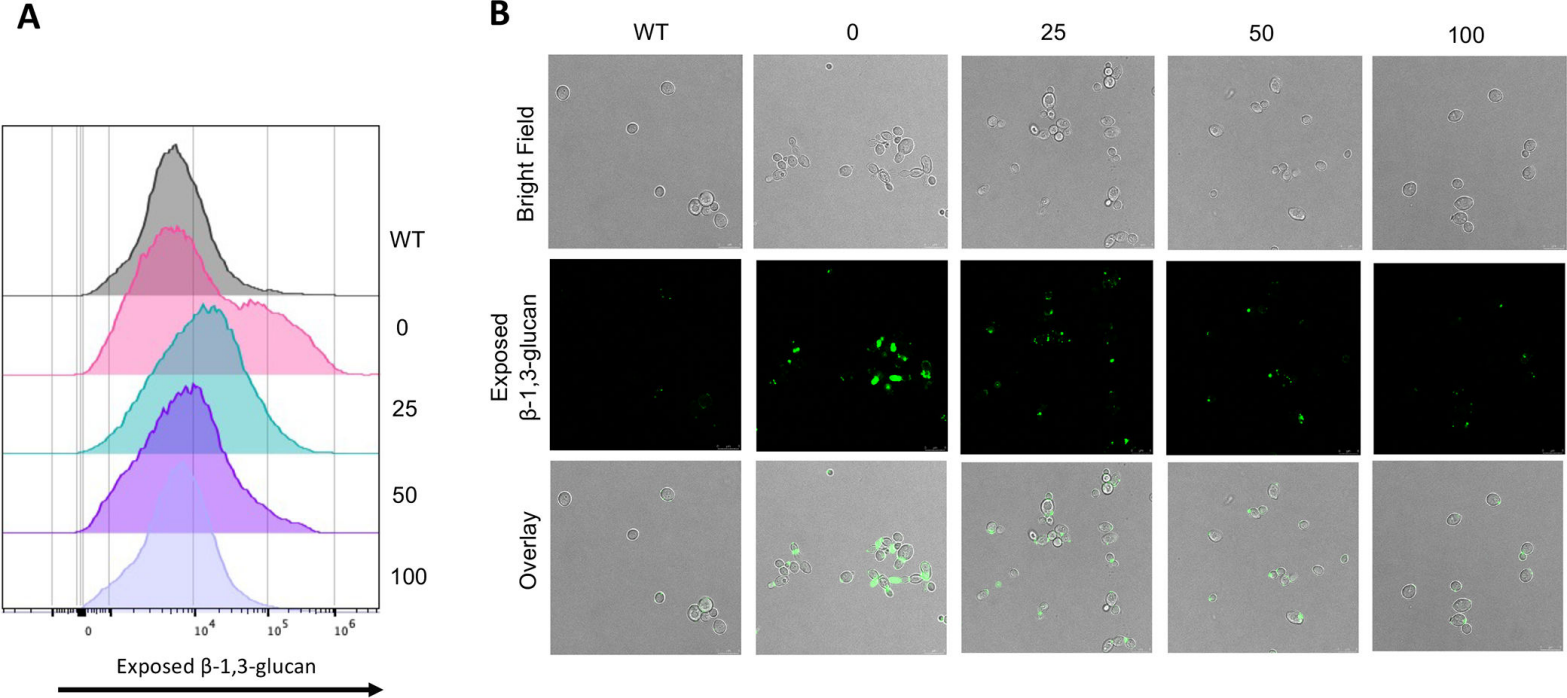
The *ura3*ΔΔ mutant is unmasked at the stationary phase. Wild-type and *ura3*ΔΔ cells were grown overnight, then diluted and grown for 48 hours in YPD with 0, 25, 50, or 100 µg/mL uridine, and stained with an anti-β-1,3-glucan antibody and an Alexa Fluor 488 conjugated secondary antibody for flow cytometry (**A**) and confocal microscopy (**B**), with a representative histogram and image shown for each sample population.

Uridine supplementation also exhibited a dose-dependent restoration in the growth of the *ura3*ΔΔ mutant compared with wild type, although its growth clearly lagged wild-type growth at all time points (Fig. 2C and D). This observation led us to question whether β-1,3-glucan exposure in the *ura3*ΔΔ mutant at the 16-hour time point is due to it being in a different stage of growth since the growth phase impacts unmasking (47). To test this, we measured β-1,3-glucan exposure in SC5314 and *ura3*ΔΔ cells grown for 48 hours rather than at the previously observed 16-hour time point to ensure that all strains were in a similar stage of growth. We observed a dose-dependent relationship between uridine supplementation and restoration of masking in the *ura3*ΔΔ mutant at the 48-hour time point (Fig. S3). The 0 µg/mL MFI was lower than the 25 µg/mL MFI, but this is likely because the 0 µg/mL population had a non-normal distribution that includes a population with very high β-1,3-glucan exposure (histograms are represented in Fig. 3A). As can be seen by microscopy, the *ura3*ΔΔ 0 µg/mL cells exhibited some daughter cells that were very strongly unmasked (Fig. 3B). In fact, the *ura3*∆∆ mutant exhibits fewer daughter cells at the 48-hour time point as the uridine concentration increases, but these daughter cells still have higher exposure of β-glucan than the mother cells. In contrast, the wild-type cells when budding in log phase (3-hour growth) exhibited a distinct pattern of β-1,3-glucan exposure compared with the *ura3*ΔΔ mutant at the 16- or 48-hour time points, with punctate unmasking on both mother and daughter cells in wild-type log phase cells, as opposed to the very strong unmaking of just the daughter cells seen in the *ura3*∆∆ mutant (Fig. S4). This indicates that this is not just a consequence of the budding stage. Since increased unmasking of the *ura3*ΔΔ mutant was observed at the 16- and 48-hour time points, we used the 16-hour time point for the remainder of experiments in this study as the *ura3*ΔΔ cells do not show major cell wall defects in this state, whereas 48-hour growth may allow for the accumulation of pleiotropic effects related to deep stationary phase.

**Fig 4:**
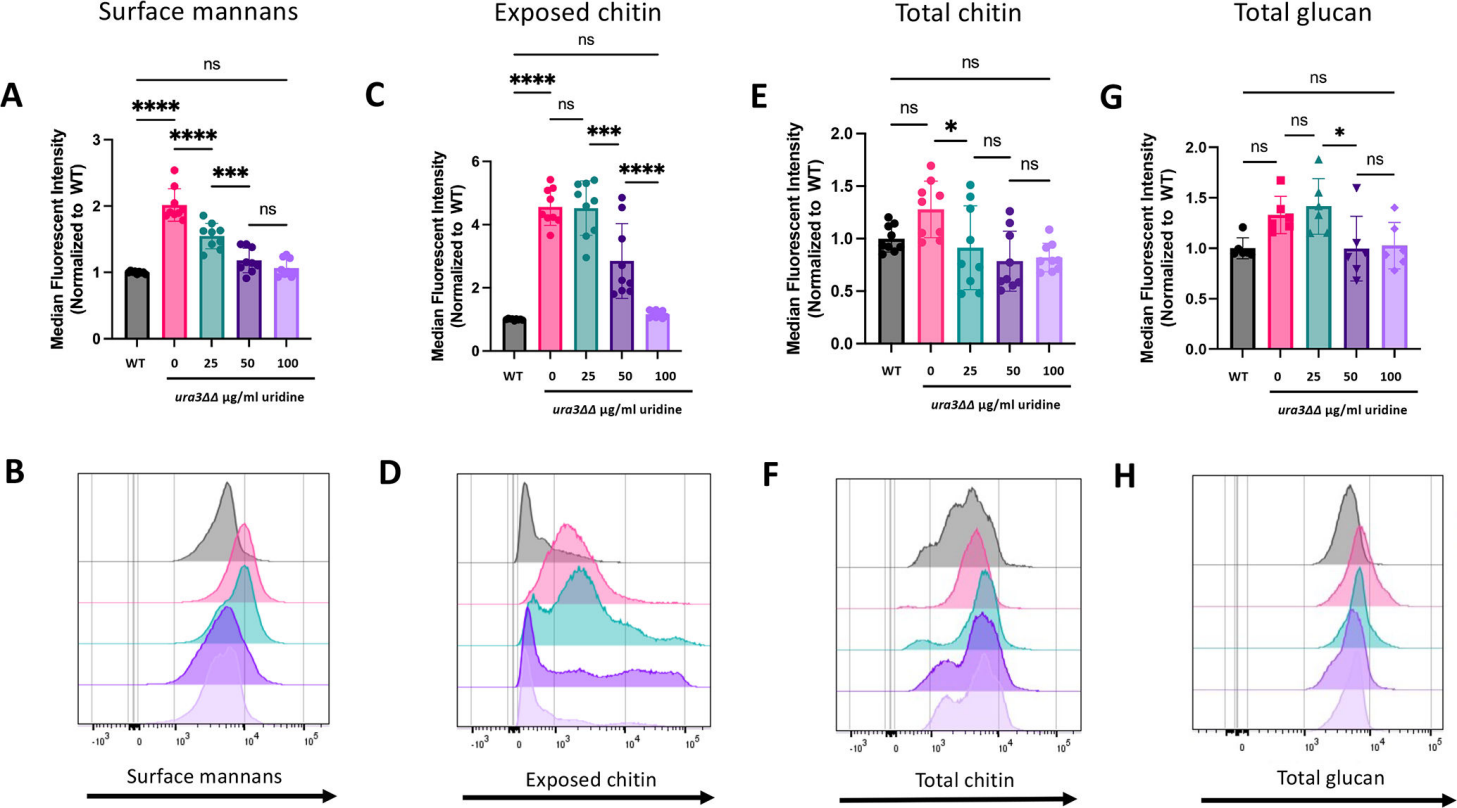
Surface mannans and exposed chitin, but not total levels of chitin or β-glucan, are altered in the *ura3*ΔΔ mutant. For all pairs of graphs, the colors in each histogram are representative of those in the associated bar graph. (**A**) Wild-type and *ura3*ΔΔ cells were grown overnight in YPD with 0, 25, 50, or 100 µg/mL uridine and stained with concanavalin A for flow cytometry, and the MFI of surface mannans was measured. (*n* = 9 biological replicates with one technical replicate for each, **P < 0.01, by one-way ANOVA.) (**B**) A representative histogram of the population stained with concanavalin A is also shown. (**C**) Cells were grown overnight in different uridine concentrations and stained with wheat germ agglutinin for flow cytometry, and the MFI of exposed chitin was measured. (*n* = 9 biological replicates with one technical replicate for each, *P < 0.05, **P < 0.01, ****P < 0.0001, by one-way ANOVA.) (**D**) A representative histogram of the population stained with wheat germ agglutinin is also shown. (**E**) Cells were grown overnight and stained with calcofluor white for flow cytometry, and the MFI of total chitin was measured. (*n* = 9 biological replicates with one technical replicate for each, ns by one-way ANOVA). (**F**) A representative histogram of the population stained with calcofluor white is also shown. (**G**) Cells were grown overnight and stained with aniline blue for flow cytometry, and the MFI of total β-glucan was measured. (*n* = 6 biological replicates with one technical replicate for each, ns by one-way ANOVA.) (**H**) A representative histogram of the population stained with aniline blue is also shown.

Previous literature has shown that chitin production may increase to compensate for decreases in β-1,3-glucan or β-1,6-glucan levels, and this correlates with unmasking (48–50). Thus, we tested whether other cell wall architectural components were disrupted in the uridine auxotroph. To do this, we used concanavalin A, wheat germ agglutinin, calcofluor white, and aniline blue dyes to measure the levels of surface mannan, exposed chitin, total chitin, and total β-glucan, respectively, in SC5314 and *ura3*ΔΔ cells with increasing concentrations of uridine. Interestingly, surface mannan levels in the *ura3*ΔΔ mutant significantly increased compared with wild type and were ~2-fold higher when no uridine was present, but subsequently decreased with increasing uridine, following a similar trend to β-1,3-glucan exposure (Fig. 4A and B). However, *ura3*ΔΔ cells exhibited an increase in median cell size compared with wild type in 0 µg/mL (Fig. S5). This size difference was ~1.1fold, indicating that cell size differences do not fully account for the increase in mannan levels.

The *ura3*ΔΔ mutant with no uridine supplementation also had the greatest amount of exposed chitin (Fig. 4C and D). Addition of 50 and 100 µg/mL uridine, but not 25 µg/mL uridine, significantly decreased the median fluorescent intensity. However, representative histograms revealed that even at 25 µg/mL uridine, a subpopulation of cells exhibited wild-type chitin exposure, and population dynamics of exposed chitin shifted toward wild-type levels in a dose-dependent manner. Surprisingly, total chitin and total glucan levels appeared not to be impacted by the lack of *de novo* uridine biosynthesis. The representative histogram of the total chitin population (Fig. 4E and F) revealed a non-normal distribution with peaks that exhibited variable chitin levels. Likewise, there were dose-dependent shifts in the chitin subpopulations at 25, 50, and 100 µg/mL uridine compared with wild type and the *ura3*ΔΔ mutant at 0 µg/mL uridine, although these were not significant based on the median fluorescent intensity. Furthermore, the level of total β-glucan was not significantly different in the uridine auxotroph with or without exogenous uridine, and the histogram of the population revealed no subpopulations (Fig. 4G and H). Thus, uridine auxotrophy appears to impact the level of surface mannans and exposed chitin, but not total chitin or total β-glucan, in the cell wall.

### UDP-glucose and UDP-*N*-acetylglucosamine levels, but not cell wall synthesis gene expression, are decreased in a uridine auxotroph

**Fig 5:**
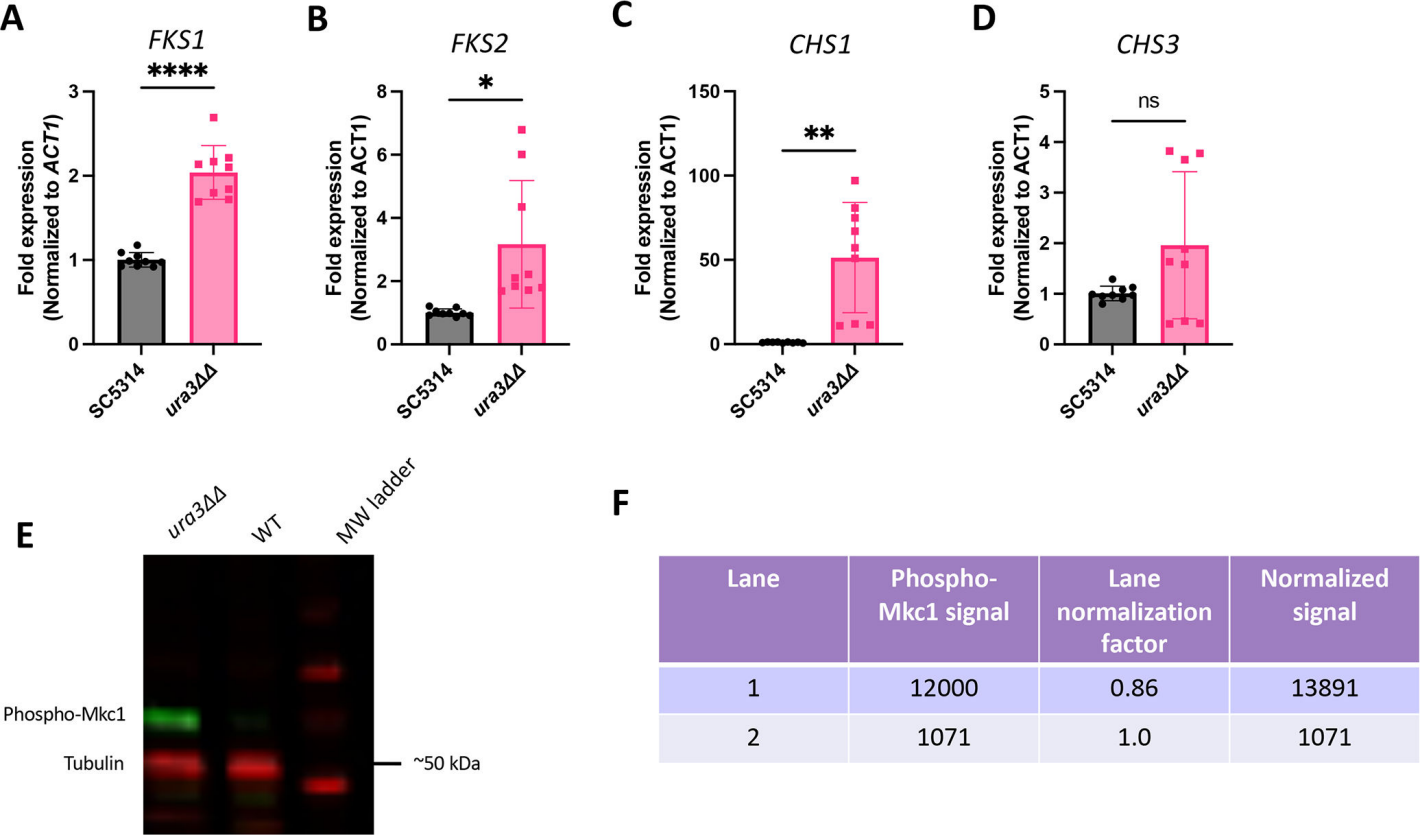
Expression of β-1,3-glucan synthase and chitin synthase genes and activation of the Mkc1 MAPK pathway are increased in the *ura3*ΔΔ mutant. (**A–D**) Wild-type and *ura3*ΔΔ cells were grown overnight (~16 hours) in YPD for RNA extraction for RT-qPCR. Relative transcript abundance of *FKS1* (**A**), *FKS2* (**B**), *CHS1* (**C**), and *CHS3* (**D**) was measured. (*n* = 3 biological replicates with three technical replicates for each, **P = 0.0055, ***P = 0.0003, ****P < 0.0001, by Welch’s *t*-test.) (**E**) Cells were grown to mid-log phase (OD_600_ = 0.8) for protein extraction, and western blotting for Mkc1 activation was assessed using an anti-p44/42 antibody and an anti-tubulin antibody for loading control. (**F**) Signal intensities of phospho-Mkc1 in each lane were measured and normalized to tubulin loading control.

β-1,3-Glucan and chitin are synthesized by enzymes encoded by the *FKS* genes and *CHS* genes, respectively (41, 51–53). These synthases require sufficient levels of precursors to produce cell wall polysaccharides. Although the exposure of β-1,3-glucan and chitin in a uridine auxotroph is significantly increased, the total levels of these components did not decrease compared with wild type. Thus, we hypothesized that the expression of β-1,3-glucan and chitin synthase genes would not be significantly altered in the *ura3*ΔΔ mutant. To test this, we measured the expression of the β-1,3-glucan synthase genes *FKS1* and *FKS2* and the chitin synthase genes *CHS1* and *CHS3* in wild-type and *ura3*ΔΔ strains using RT-qPCR. Contrary to our hypothesis, we found that the expression of the *FKS* genes significantly increased in the *ura3*ΔΔ mutant compared with wild type (Fig. 5A and B). The *ura3*ΔΔ mutant also exhibited greatly increased expression of *CHS1*; however, no clear trend in expression could be determined for *CHS3* (Fig. 5C and D). Since we have shown that the *ura3*ΔΔ mutant grows slower than wild type and is actively budding at the 16-hour time point, we also measured the expression of *FKS1*, *FKS2*, *CHS1*, and *CHS3* at 48 hours when the cells were in deep stationary phase. The expression trends of these genes at 48 hours largely followed those observed at 16 hours, with the exception of *CHS3* (Fig. S6). Furthermore, the GTPase Rho1 regulates Fks1 activity and has been shown to activate signaling through cell wall integrity pathways (54, 55). Thus, we measured the activation of the Cek1 and Mkc1 MAP kinase cell wall integrity pathways in wild type and the *ura3*ΔΔ mutant. The uridine auxotroph exhibited increased activation of the Mkc1 MAP kinase pathway (Fig. 5E and F). Activation of this pathway was inversely correlated with uridine supplementation (Fig. S6).

**Fig 6:**
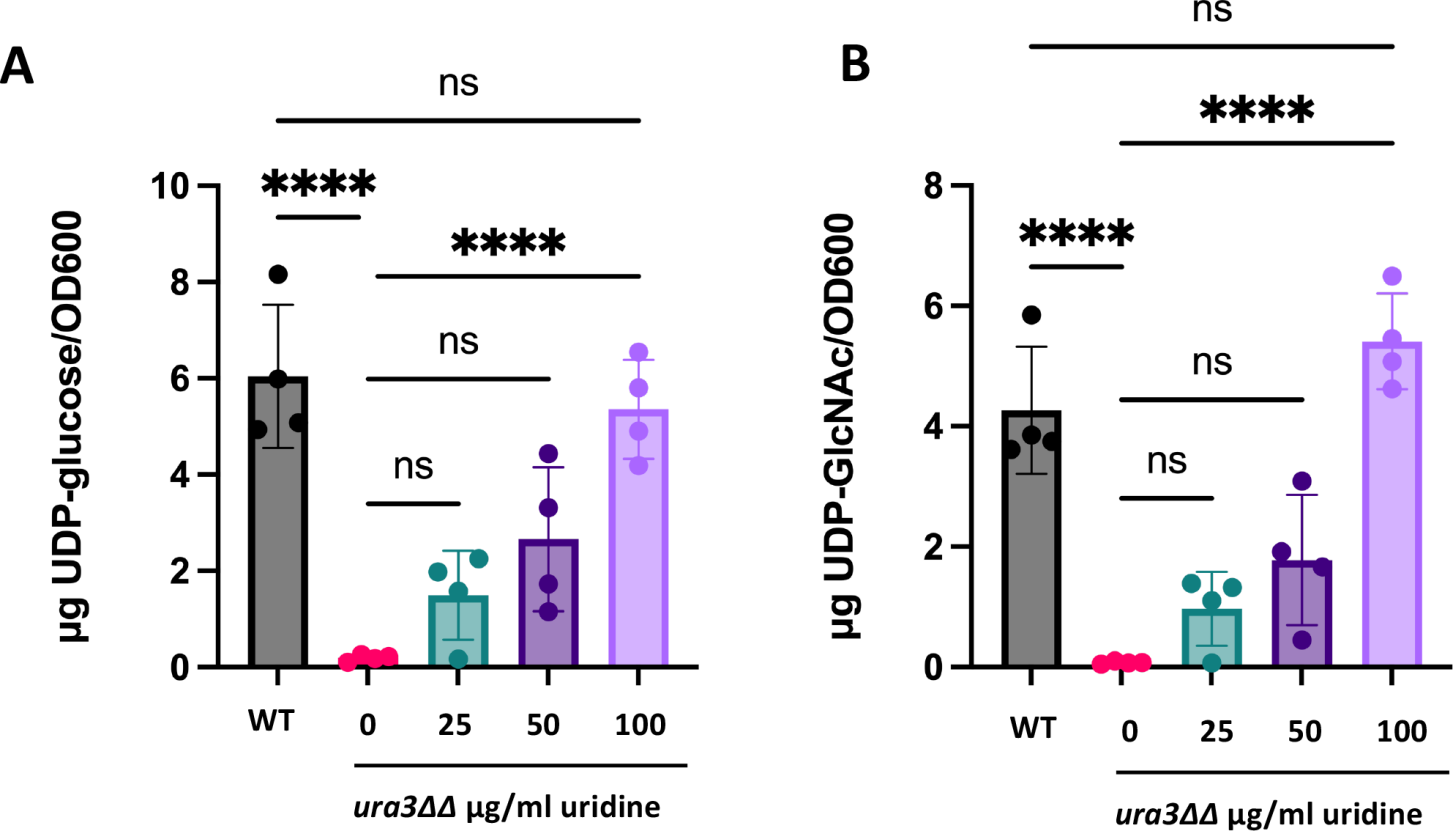
UDP-sugar levels are decreased in the *ura3*ΔΔ mutant and are rescued with increasing uridine supplementation. Wild-type and *ura3*ΔΔ mutant cells were grown overnight in YPD with 0, 25, 50, or 100 µg/mL uridine and analyzed by LC-MS/MS for quantification of (**A**) UDP-glucose and (**B**) UDP-*N*-acetylglucosamine. (*n* = 4 biological replicates with one technical replicate for each, ****P < 0.0001, by one-way ANOVA.)

We have shown that the deposition of cell wall components is altered when *C. albicans* is uridine limited. However, it is unclear whether the observed changes to cell wall composition are driven by insufficient levels of UDP-sugars necessary to build cell wall polysaccharides. To test this, we quantified UDP-glucose and UDP-GlcNAc in wild-type and *ura3*ΔΔ cells grown in YPD with 0, 25, 50, or 100 µg/mL uridine by liquid chromatography-mass spectrometry (LC-MS/MS). The amount of UDP-glucose decreased in the *ura3*ΔΔ mutant with no uridine supplementation compared to wild type and increased in a dose-dependent manner (Fig. 6A). UDP-GlcNAc levels in wild type and the *ura3*ΔΔ mutant followed a similar trend to UDP-glucose levels (Fig. 6B). The same trend for both UDP-glucose and UDP-GlcNAc levels was observed in additional biological replicates (Fig. S7). In contrast, the levels of GDP-mannose, the precursor to mannan, were not significantly altered between any of the conditions, and no clear trend was observed (Fig. S8). This supports the hypothesis that the *ura3*ΔΔ mutant is deficient at synthesizing UDP-sugars and that this deficiency can be restored with uridine supplementation.

### Attenuation in virulence of uridine auxotrophs is not completely dependent on the host immune system

**Fig 7:**
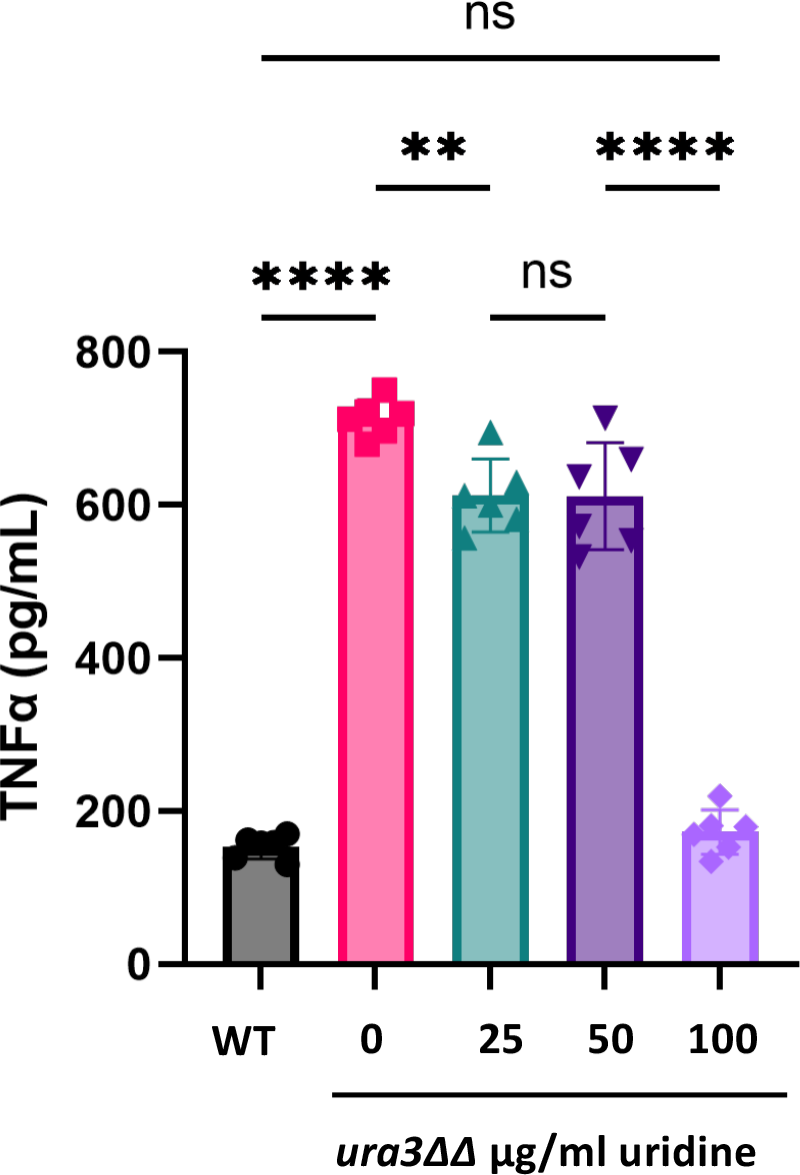
RAW264.7 macrophages are activated by the *ura3*ΔΔ mutant in a uridine dose-dependent manner. Wild-type and *ura3*ΔΔ mutant cells were co-incubated with RAW264.7 macrophages, and TNFα release from macrophages was measured by enzyme-linked immunosorbent assay (ELISA). (*n* = 6 biological replicates with one technical replicate for each, **P < 0.01, ****P < 0.0001, by one-way ANOVA.)

Previous literature has shown that the CAI-4 uridine auxotrophic strain is avirulent in a murine model of systemic infection (43). This avirulence is thought to be due to uridine auxotrophy decreasing the growth of *C. albicans* in the host. Our finding that uridine auxotrophs exhibit increased β-1,3-glucan exposure led us to question the role of the host’s immune system in virulence attenuation. We hypothesized that uridine auxotrophs are attenuated in virulence due to increased exposure of β-1,3-glucan driving host immune recognition and clearance of the fungus. Thus, we assessed the extent to which the *ura3*ΔΔ mutant elicits TNFα secretion from RAW264.7 murine macrophages *in vitro*. Co-incubation of *ura3*ΔΔ mutant cells with murine macrophages revealed an inverse correlation between TNFα secretion and uridine concentration (Fig. 7). This trend closely followed that of exposed β-1,3-glucan in the *ura3*ΔΔ mutant at 16 hours. These results suggested that immune cells may more efficiently recognize and clear the uridine auxotroph, thus warranting further investigation *in vivo*.

**Fig 8:**
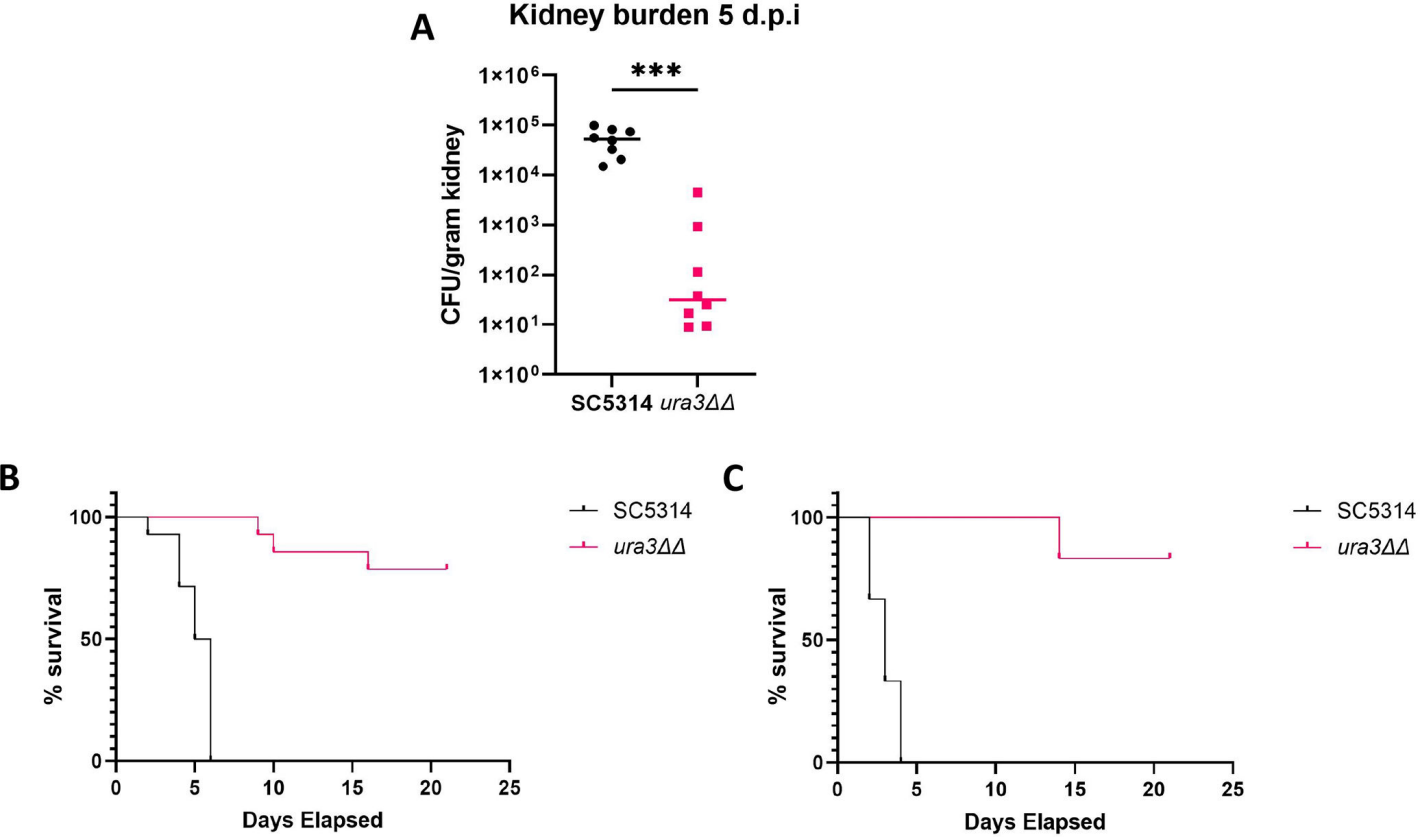
Attenuation in virulence of the *ura3*ΔΔ mutant is not dependent on the host immune system. (**A**) Institute of Cancer Research (ICR) mice were infected intravenously with 1 × 10^6^ wild-type or *ura3*ΔΔ cells, and kidneys were harvested 5 d.p.i and measured for fungal burden (*n* = 8 mice for each strain, ***P = 0.0002, by student’s *t*-test). (**B**) ICR mice were infected intravenously with 1 × 10^6^ cells of wild-type or *ura3*ΔΔ strains and were monitored for survival over a 21-day period. (*n* = 14 mice for each strain). (**C**) ICR mice were immunosuppressed with 150 mg/kg cyclophosphamide injected intraperitoneally 4 days prior to infection, continuing with injections every third day. Mice were injected intravenously with 1 × 10^3^ cells of wild-type or *ura3*ΔΔ strains and monitored for survival over a 21-day period. (*n* = 6 mice per strain.)

We performed systemic murine infections with wild-type or *ura3*ΔΔ strains. We infected immunocompetent mice intravenously with 1 × 10^6^ cells and measured kidney fungal burden 5 days post-infection (d.p.i.). As expected, mice infected with the *ura3*ΔΔ mutant had significantly decreased kidney fungal burden 5 d.p.i. (Fig. 8A). To determine whether the reduced fungal burden is due to increased exposure to immunogenic polymers in the cell wall, we challenged immunocompetent and immunosuppressed mice with wild-type or *ura3*ΔΔ cells and monitored their survival over 21 days. Immunocompetent mice were infected intravenously with 1 × 10^6^ cells, and immunosuppressed mice were treated with cyclophosphamide, a broad-spectrum immunosuppressant, and then infected intravenously with 1 × 10^3^ cells (56). The survival of the *ura3*ΔΔ infected immunocompromised mice closely followed the trend of survival of the immunocompetent mice infected with *ura3*ΔΔ cells, where mice infected with the *ura3*ΔΔ mutant showed enhanced survival in both conditions (Fig. 8B and C). This indicates that the attenuated virulence of the *ura3*ΔΔ mutant is not driven by increased β-1,3-glucan exposure. Rather, uridine auxotrophy renders the fungus unfit within the host, most likely through reduced growth rate.

**Fig 9:**
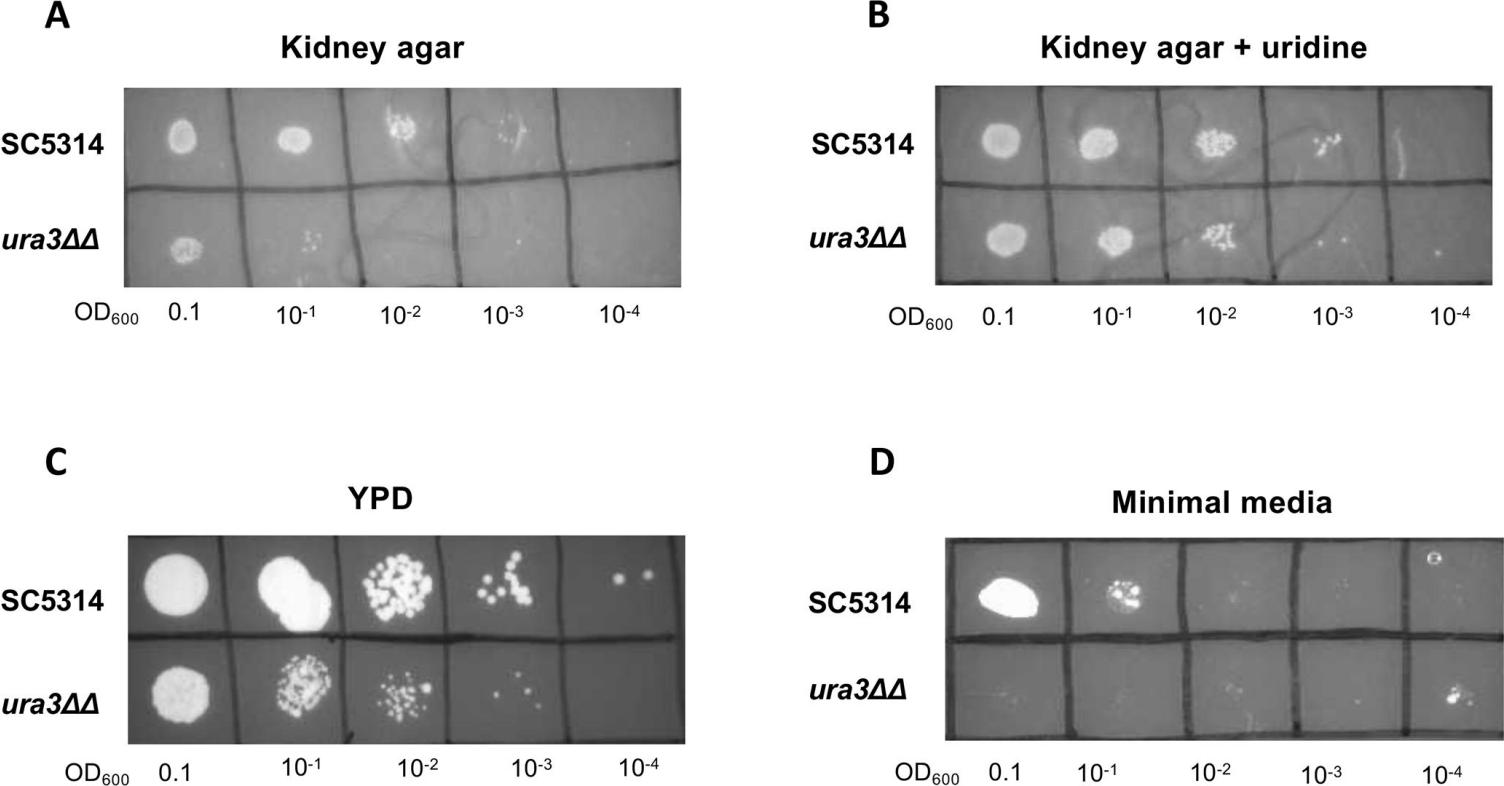
Kidney nutrients are not sufficient for the growth of the *ura3*ΔΔ mutant. (**A**) Kidneys were harvested from uninfected mice to make kidney agar plates. Wild-type and *ura3*ΔΔ mutant cells were grown overnight in YPD and spot diluted onto the kidney agar. (**B**) Spot dilution of wild type and the *ura3*ΔΔ mutant on kidney agar with 100 µg/mL uridine plated on top. (**C**) Spot dilution of wild type and the *ura3*ΔΔ mutant on YPD (with no uridine supplementation) as a nutrient-rich control. (**D**) Spot dilution of wild type and the *ura3*ΔΔ mutant on minimal media as a uridine-depleted control.

Previous literature has measured the uridine concentration in the plasma of mammals to be ~5 µM (~1.2 µg/mL) (57). However, it is unknown whether the kidneys contain sufficient uridine to support the growth of uridine auxotrophs. Thus, we developed an *ex vivo* kidney agar assay to determine whether the *ura3*ΔΔ mutant can survive on kidney nutrients. We harvested the kidneys of healthy, uninfected mice and then homogenized them, filtered the extract, and mixed it with agar to create kidney agar plates. A spot dilution assay of wild-type and *ura3*ΔΔ cells onto kidney agar showed that wild type grows similarly on YPD and kidney agar, whereas the *ura3*ΔΔ mutant grows significantly worse on kidney agar than on YPD (Fig. 9A and C). The growth defect of the *ura3*ΔΔ mutant on kidney agar was rescued by supplementation with 100 µg/mL uridine into the media (Fig. 9B). Each strain was also spot diluted onto minimal media lacking uridine as a control (Fig. 9D). Thus, it appears that the kidney is limited in available uridine, which compromises the growth of mutants with uridine auxotrophy.

## DISCUSSION

We have shown that a uridine auxotroph exhibits increased β-1,3-glucan and chitin exposure as well as increased surface mannans compared with wild type (Fig. 1A, 4A and B). These phenotypes are rescued in a dose-dependent manner with exogenous uridine. Interestingly, total levels of chitin and β-glucan do not decrease in a uridine auxotroph (Fig. 4E through H). Previous literature has shown that uridine auxotrophy attenuates virulence in a murine model of systemic infection (43). Here, we show that although a uridine auxotroph exhibits cell wall architectural changes that expose immunogenic polymers, this is not the main driver of virulence attenuation. Similar survival trends were observed between immunocompetent mice and immunosuppressed mice challenged intravenously with a uridine auxotroph (Fig. 8B and C). Furthermore, a uridine auxotroph is unable to readily grow on kidney agar, suggesting that the *ura3*ΔΔ mutant exhibits severely decreased growth in the host (Fig. 9A through C). Although it is possible that increased immunogenicity does contribute to fungal clearance, this is overshadowed by growth defects.

**Fig 10:**
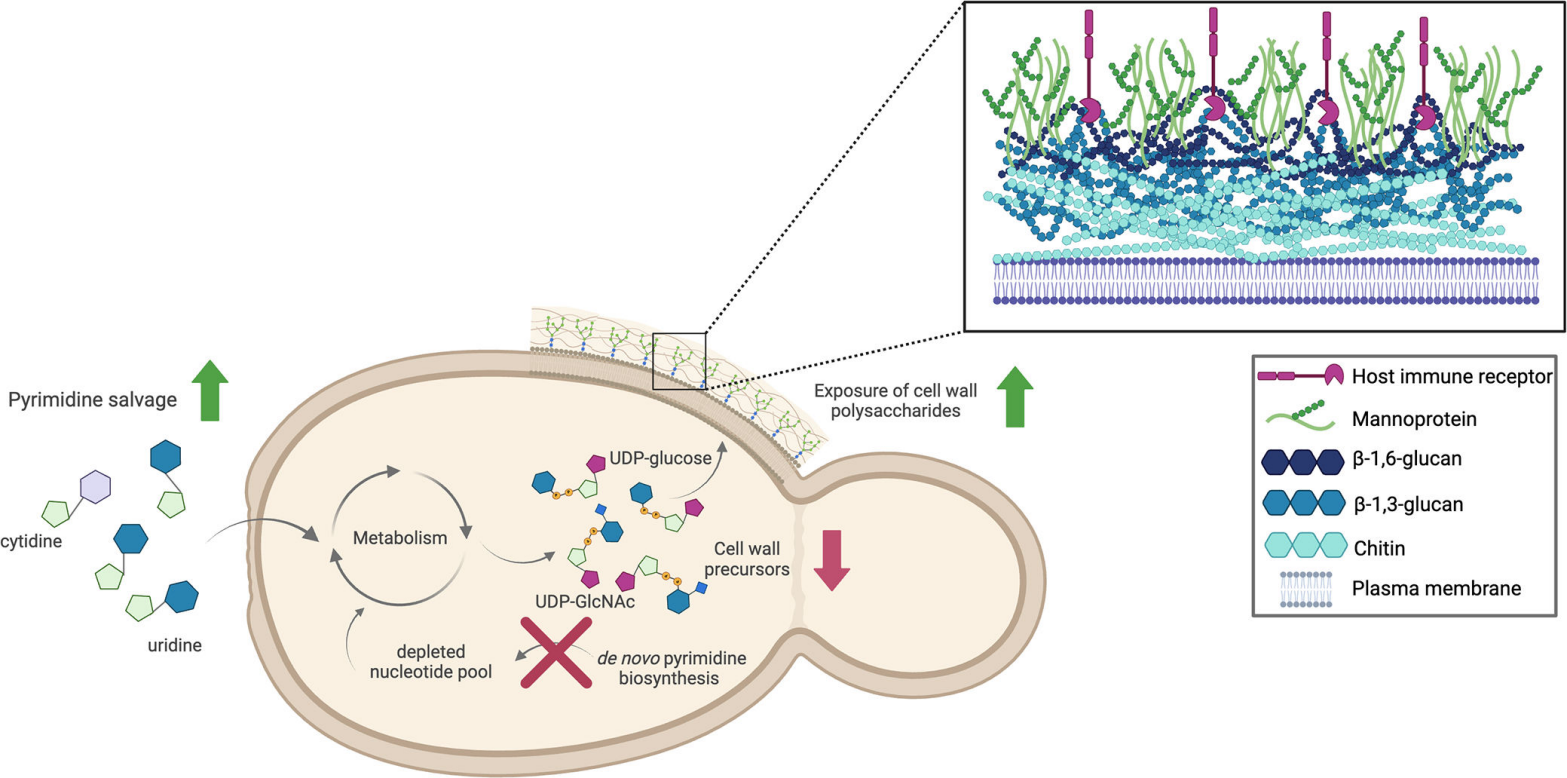
Proposed model for pyrimidine acquisition and its effect on cell wall architecture in a uridine auxotroph. Schematic showing the proposed effects of disruption to *de novo* pyrimidine biosynthesis on the pyrimidine salvage pathway, cell wall precursors, and cell wall polysaccharides. Figure created with BioRender.

In wild-type *C. albicans*, both the *de novo* pyrimidine biosynthetic and pyrimidine salvage pathways produce uridine for use in various aspects of metabolism including RNA and cell wall synthesis. As in *S. cerevisiae*, *C. albicans* likely prefers the pyrimidine salvage pathway to acquire uridine. However, the acquisition of sufficient uridine from this pathway to feed into metabolism is environmentally dependent (30). Phosphorylated forms of uridine acquired from these pathways are used along with sugars from central carbon metabolism to create UDP-glucose and UDP-GlcNAc. Cell wall components are then made from these UDP-sugar precursors to build the cell wall. In a uridine auxotroph, the *de novo* pyrimidine biosynthetic pathway is nonfunctional; thus, the cell must rely solely on pyrimidine salvage (Fig. 10). If the cell cannot acquire sufficient exogenous uridine for metabolism, the levels of UDP-sugars decrease. The cell wall architecture is altered in response, and the levels of exposed β-1,3-glucan and chitin and surface mannans increase.

Surprisingly, despite the observation that the uridine auxotroph has decreased levels of UDP-glucose and UDP-*N*-acetylglucosamine, as predicted, along with increased exposure of β-1,3-glucan and chitin, the total levels of these polymers remain the same. There are several plausible explanations for this observation. We show that a uridine auxotroph exhibits increased *FKS1*, *FKS2*, and *CHS1* expressions (Fig. 5A through D). It is possible that increased expression of β-1,3-glucan and chitin synthases attempts to compensate for decreased precursors and ultimately prevents cell wall polymer levels from decreasing as well. It is also possible that the decreased levels of precursor being delivered to the β-1,3-glucan synthase causes the cell to behave as if Fks1 is being inhibited, especially if more enzyme is produced with insufficient amounts of substrate. This effect on the cell could mirror that of caspofungin treatment, which inhibits β-1,3-glucan synthase, increases β-1,3-glucan exposure, increases chitin levels, and activates the Mkc1 stress response pathway (34, 35, 58–60). Although this model is plausible, it neither explains why the amount of total chitin does not change in the uridine auxotroph nor does it explain the apparent biphasic effects on exposed chitin and total chitin as uridine concentration is increased. Caspofungin treatment increases chitin synthesis in the cell, likely as a compensation mechanism (61, 62). Chitin deposition in response to caspofungin treatment is biphasic in flow cytometry experiments when cells are inhibited for Mkc1 or calcineurin activity (58). Thus, if the uridine auxotroph is undergoing a caspofungin-like stress response, this may explain these biphasic changes. Total chitin levels do not increase in *ura3*∆∆ mutant; however, the loss of UDP-GlcNAc may preclude this response. It is also plausible that the biphasic exposed chitin and total chitin populations are simply due to the absence of sufficient uridine to drive the entire population toward wild-type levels, resulting in a population with an intermediate phenotype.

Another surprising phenotype of the uridine auxotroph was the increase in surface mannans in the cell wall, given that GDP-mannose levels are not significantly changed. Although we found that the uridine auxotroph population without uridine supplementation has a greater median cell size than the wild-type population, we hypothesize that this does not fully explain the ~2-fold increase in surface mannans. It is possible that this increase in mannans may be due to a compensation mechanism, akin to chitin during caspofungin treatment, but there are few papers characterizing the potential role of mannan compensation (63, 64). Another plausible explanation is that the uridine auxotroph slows growth so that cells lacking in precursors can still maintain cell wall integrity. In this model, the uridine auxotroph incorporates uridine into UDP-sugars for cell wall synthesis as it becomes available, since most of the salvaged uridine is likely prioritized toward RNA synthesis. This results in a cell wall with a non-uniform distribution of cell wall components, particularly the mannans, as their incorporation into the cell wall is predicated on attachment to β-glucans. This may cause pockets of thicker mannan attachment and pockets where their distribution is not as dense, allowing exposure of β-1,3-glucan and chitin. Importantly, although the stains used to quantify the different cell wall components via flow cytometry in Fig. 4 are relatively simple, quick, and cost-effective, they are not entirely specific to the cell wall components of interest and do not provide as thorough of a compositional analysis as biochemical measurements would. The stains also do not reveal any information about the spatial arrangement of mannan in the cell wall. Thus, further investigation is necessary to fully understand the cell wall architecture in the uridine auxotroph.

Finally, the cell wall architecture of the *ura3*ΔΔ mutant lacking exogenous uridine is unique compared with many other conditions when examined at 48 hours. Cells seem to arrest in a budded form but have extremely high levels of β-1,3-glucan exposure, contrary to wild-type cells actively budding. This may be a consequence of depleting necessary cell wall precursors necessary for growth in a uridine-limited environment; however, further investigation is needed to understand this process.

## MATERIALS AND METHODS

### Growth media and culture conditions

*C. albicans* strains (Table S3) were grown in YPD medium (1% yeast extract, 2% peptone, 2% dextrose) (Thermo Fisher Scientific) or minimal medium (0.67% yeast nitrogen base without amino acids, 2% dextrose) (Thermo Fisher Scientific) shaking at 225 rpm at 30°C (65). For cells grown with exogenous uridine, YPD broth was supplemented with the indicated amount of uridine (Sigma) before autoclaving. RAW264.7 macrophages were grown in Dulbecco’s modified Eagle medium (DMEM) with L-glutamine (Gibco) with the addition of 10% fetal bovine serum (Invitrogen) and 1% penicillin-streptomycin (Thermo Fisher Scientific) at 37°C with 5% CO_2_ (66).

### Strain construction

CRISPR-Cas9 gene editing was used to create the *ura3*ΔΔ mutation in the SC5314 background using *in vitro* assembled ribonucleoprotein complexes as previously described (67). The disruption repair templates were amplified with the primers URA3CC9KO-F and URA3CC9KO-R using linearized pBSS2 (ON246334) and CaHygB-flipper (ON287367) plasmids as templates, which harbor nourseothricin and hygromycin resistance cassettes, respectively. Correct genomic integration and excision of the resistance cassettes were confirmed via growth on uridine-supplemented YPD, YPD  + 200  µg/mL nourseothricin, and YPD  + 600  µg/mL hygromycin plates, followed by PCR confirmation. The suspected *ura3*ΔΔ strain was streaked onto a minimal medium without uridine to confirm the lack of robust growth.

The CAI4 *ura3::URA3* strain was created by amplifying the SC5314 *URA3* open reading frame (ORF) with 500 base pairs of flanking homology on the 5′ and 3′ ends using primers MMO1 and MMO2. This fragment was then transformed into CAI-4 via electroporation. Successful transformants were selected for on minimal medium.

### Cell wall staining for flow cytometry and confocal microscopy

*C. albicans* cells were stained as described previously (47, 58, 68). For 16-hour staining, cultures were grown shaking at 225 rpm at 30°C overnight. The following morning, cells were diluted to OD_600_ 0.5 in PBS or were diluted to OD_600_ 0.1 in fresh YPD and grown for 48 hours for stationary phase staining.

### Growth curves

*C. albicans* strains were grown shaking at 225 rpm at 30°C overnight in 5 mL of YPD with 0, 25, 50, or 100 µg/mL of uridine or 100 µg/mL cytidine, guanosine, or adenosine. The following morning, cells were diluted to OD_600_ 0.1 in 5 mL of fresh YPD with the same concentration of uridine used in each respective overnight culture. Growth was measured every 2 hours for the first 10 hours of growth, as well as at 24 and 48 hours. Three biological replicates were used for each strain. Growth kinetics and exponential (Malthusian) growth were determined using GraphPad Prism v9.4c.

### Measurement of gene expression

For gene expression measurements, cells were grown overnight (~16 hours) or for 48 hours, then reverse transcription-quantitative PCR (qRT-PCR) was performed as described previously (69).

### Western blot analysis of phosphorylated Mkc1

Phospho-Mkc1 levels were measured as described previously (68). Signal quantification was measured via densitometry in ImageJ, and the lane normalization factor was calculated by dividing the tubulin signal in each lane by the highest tubulin signal observed. From this, the normalized signal was calculated by dividing the Mkc1 signal in each lane by the lane normalization factor.

### Metabolomics

*C. albicans* strains were grown shaking at 225 rpm at 30°C overnight in 5 mL of YPD with 0, 25, 50, or 100 µg/mL of uridine. Five milliliters of 75% acetonitrile/25% water containing 0.1% formic acid were added to each sample for the extraction. Samples were vortexed and then centrifuged to pellet cell debris. The supernatant was transferred to a new tube, and the solvent was evaporated to dryness using a speed-vac. Samples were then reconstituted in 1 mL of 80% acetonitrile/20% water, diluted 1:10, and then analyzed using a Waters Xevo G2-XS QTof mass spectrometer interfaced with a Waters Acquity UPLC. Data processing was performed using Masslynx software. An external standard curve was made using authentic UDP-glucose, UDP-GlcNAc, and GDP-mannose standards (Sigma). Normalized amounts of UDP-glucose and UDP-GlcNAc, and GDP-mannose were calculated for each sample by dividing the μg metabolite by its respective OD_600_.

### TNFα enzyme-linked immunosorbent assay

Cells were incubated overnight in YPD with 0, 25, 50, or 100 µg/mL uridine shaking at 225 rpm at 30°C. RAW264.7 macrophages were prepared and co-incubated with UV-inactivated *C. albicans* and then measured for TNFα release via ELISA as previously described (68).

### Mouse model of systemic infection

*C. albicans* strains were grown in 50 mL cultures of YPD overnight prior to infection shaking at 225 rpm at 30°C. The following morning, cells were pelleted at 3,500 rpm for 5 minutes, washed twice with PBS, and counted on a hemocytometer. For immunocompetent mice, cells were resuspended at 1 × 10^7^ in 10 mL PBS. For immunosuppressed mice, cells were resuspended at 1 × 10^4^ in 10 mL PBS. Mice were intravenously injected through the lateral tail vein with 100 µL of the resuspension. A portion of the immunocompetent mice was euthanized at 5 days post-infection, and their kidneys were harvested to measure kidney fungal burden. Preparation of kidneys, plating for fungal burden, and statistical analysis were performed as previously described (68). The remaining mice were monitored for survival over 21 days. Outbred ICR mice were used for all experiments (ENVIGO).

### Immune cell depletion in mice

Mice were treated intraperitoneally with 150 mg/kg cyclophosphamide (Sigma-Aldrich) as previously described (68). Immunosuppressed mice were administered cyclophosphamide 4 days prior to infection followed by injections every third day until euthanized.

### Kidney agar plates

Kidneys were harvested from uninoculated C57BL/6 mice that were designated to be culled from a colony and placed in 1× 3-(N-morpholino)propanesulfonic acid (MOPS) buffer. To remove extra fat from the kidneys, cell strainers were placed in 50 mL conical tubes, kidneys were crushed to homogenize, and the homogenate was washed twice with 4 mL of 1× MOPS buffer. The resulting 8 mL of kidney homogenate solution was added to petri dishes. Then, 8 mL of 4% agar (2% final volume) with 75 µg/mL chloramphenicol was poured into the plates and swirled immediately to mix. For kidney agar with added uridine, a 100 µg/mL uridine solution (dissolved in ddH_2_O) was made, filtered, and then spread across the top of the solidified kidney agar plate. All *C. albicans* strains streaked or diluted onto the kidney agar were incubated overnight at 37°C.

## SUPPLEMENTAL MATERIAL

Supplemental Figures msphere.00287-24-s0001.pdf Figures S1-S8.

Legends msphere.00287-24-s0002.docx Legends for supplemental material.

Table S1 msphere.00287-24-s0003.docx Plasmids.

Table S2 msphere.00287-24-s0004.docx Primers.

Table S3 msphere.00287-24-s0005.docx Strains.

ASM does not own the copyrights to Supplemental Material that may be linked to, or accessed through, an article. The authors have granted ASM a non-exclusive, world-wide license to publish the Supplemental Material files. Please contact the corresponding author directly for reuse.
