# Supplementary material for: Disruption to de novo uridine biosynthesis alters β-1,3-glucan masking in Candida albicans
Source: mSphere. 2024 Aug 8;9(9):e00287-24. doi: 10.1128/msphere.00287-24 (PMC11423711; doi:10.1128/msphere.00287-24)
Supplement: Supplemental Figures — Figures S1-S8. [file msphere.00287-24-s0001.pdf]

A

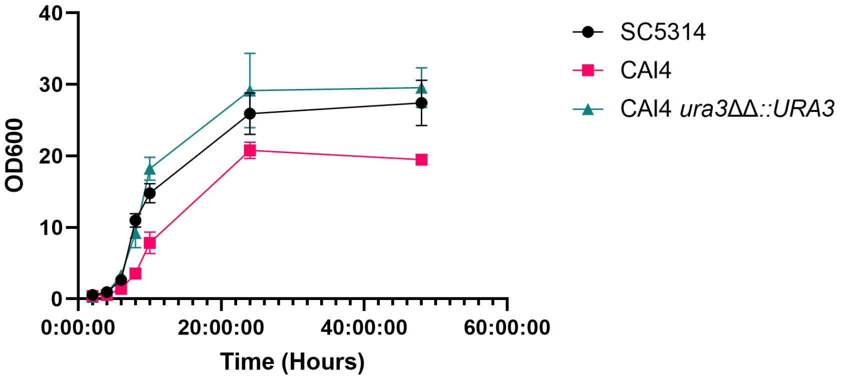

B

Sample	Doubling Time (Minutes)	95% Confidence Interval
SC5314	81.65	78.64 to 85.69
CAI-4	95.10	92.72 to 97.92
CAI-4 *ura3ΔΔ::URA3*	79.26	77.59 to 81.22

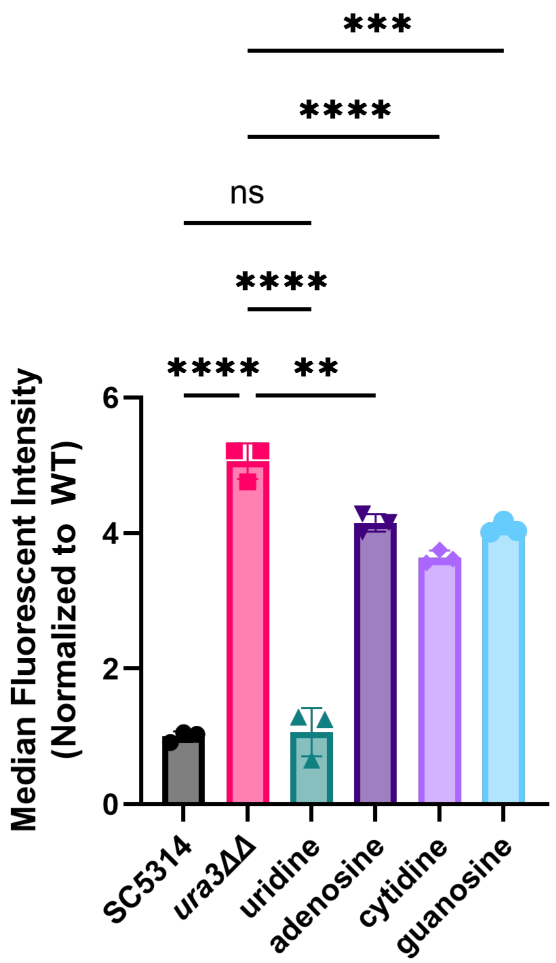
***
****
ns
****
****
**
Median Fluorescent Intensity
(Normalized to WT)
6
4
2
0
SC5314
ura3ΔΔ
uridine
adenosine
cytidine
guanosine

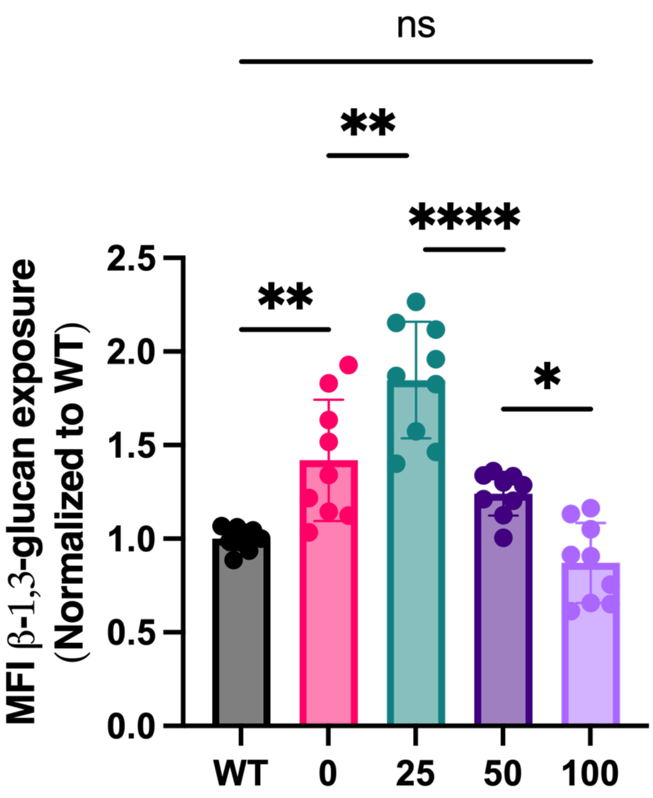

ns
**
****
**
*
MFI β-1,3-glucan exposure (Normalized to WT)
2.5
2.0
1.5
1.0
0.5
0.0
WT
0
25
50
100

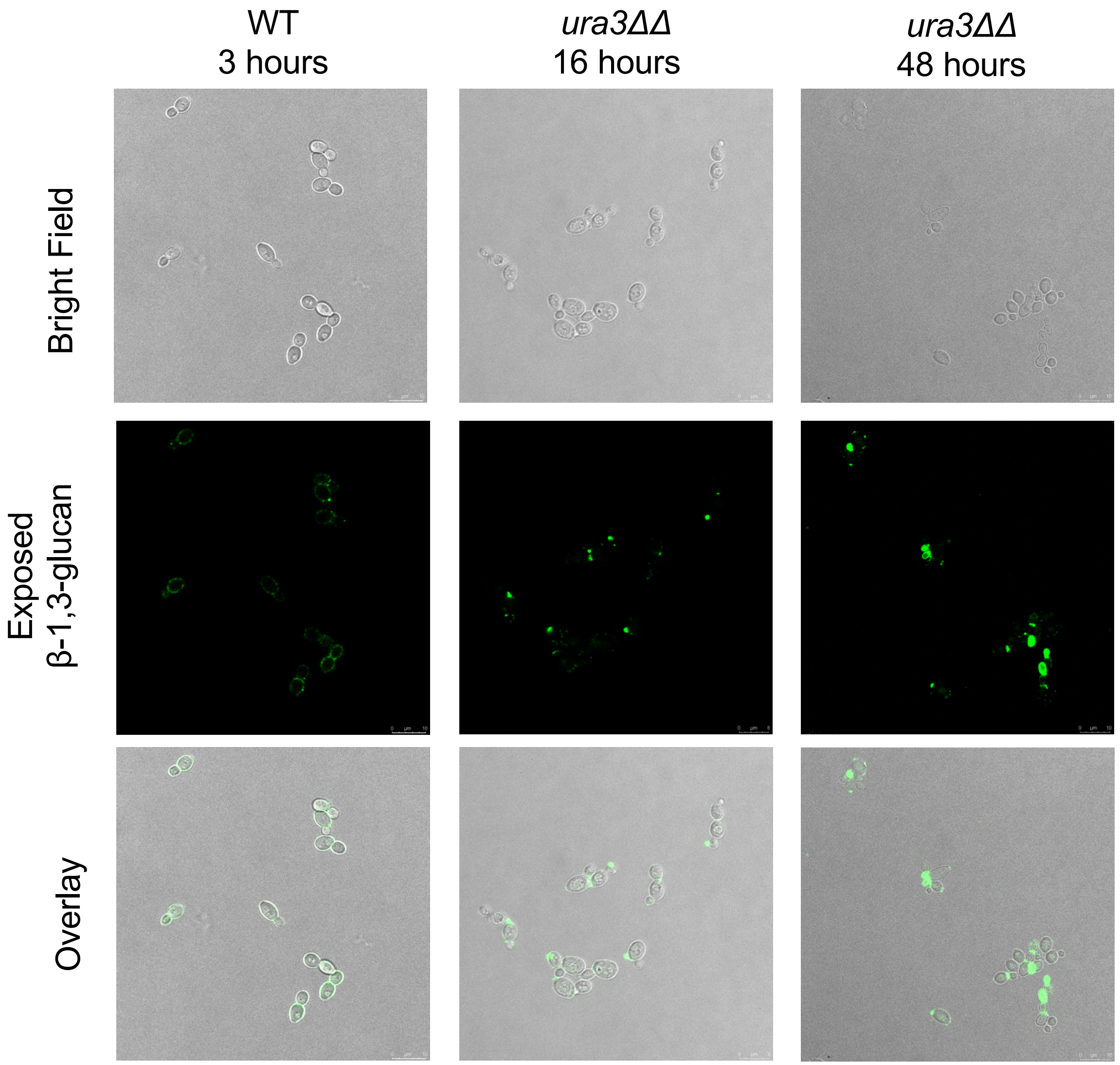

WT
3 hours
ura3ΔΔ
16 hours
ura3ΔΔ
48 hours
Bright Field
Exposed
β-1,3-glucan
Overlay

A

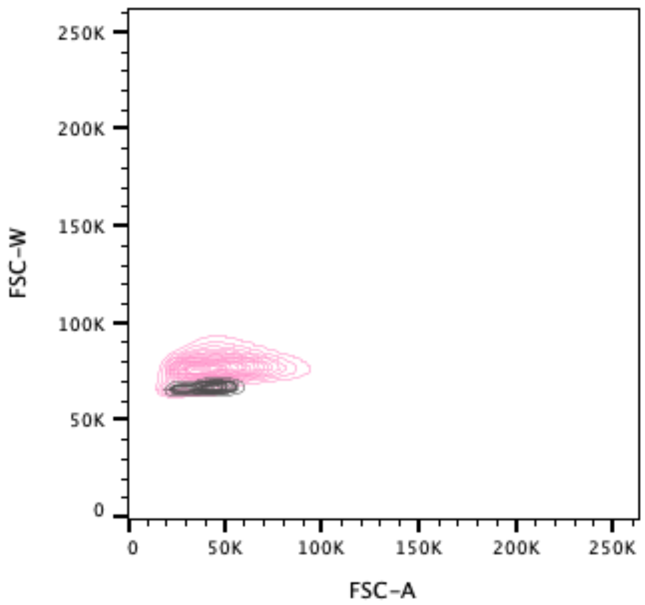
250K
200K
150K
100K
50K
0
FSC-W
0
50K
100K
150K
200K
250K
FSC-A

B

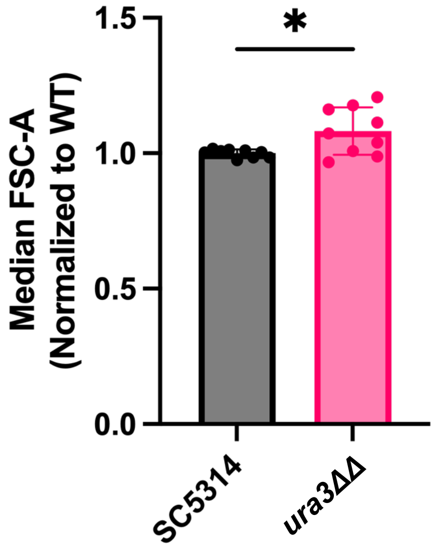
1.5
1.0
0.5
0.0
*
Median FSC-A
(Normalized to WT)
SC5314
ura3ΔΔ

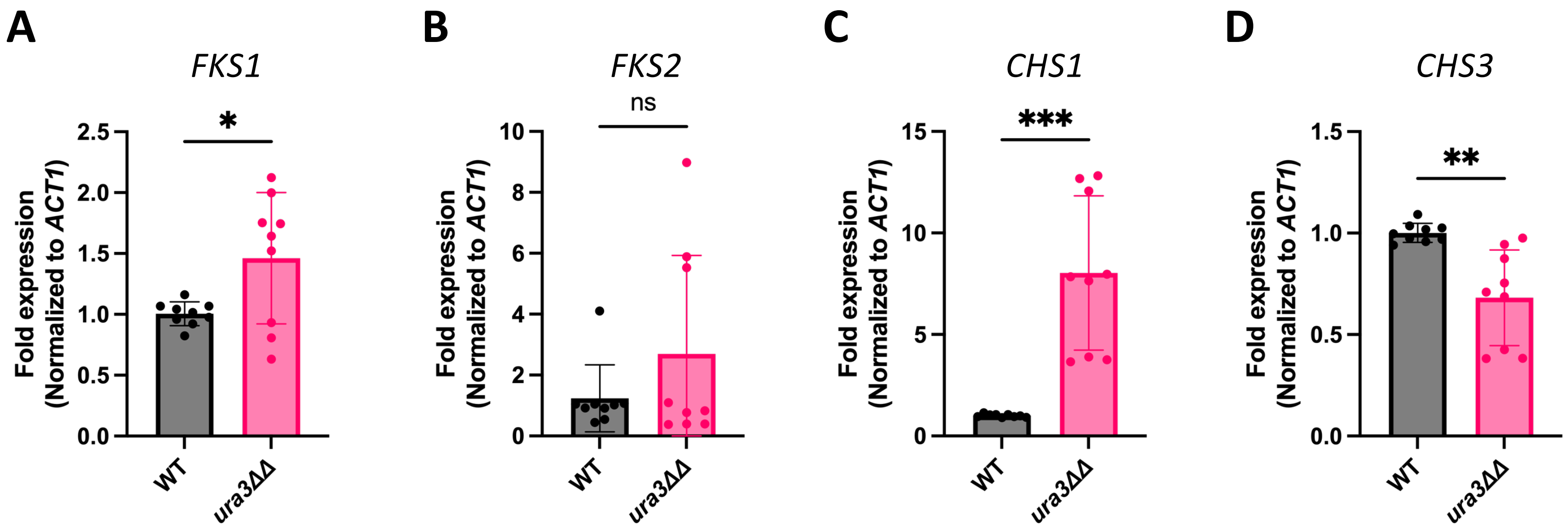

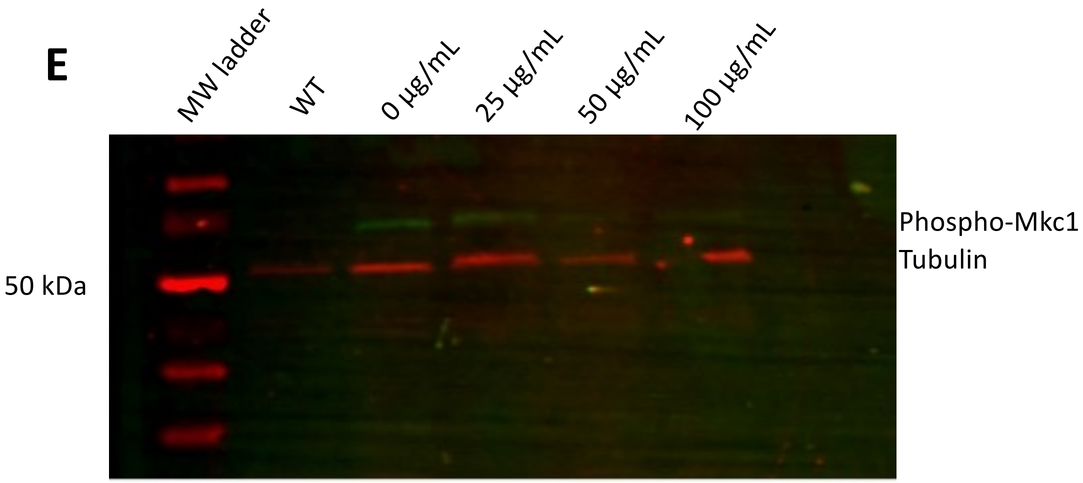

F

Lane	Phospho-Mkc1 signal	Lane normalization factor	Normalized signal
1	0	0.18	0
2	4430	1.0	4431
3	3716	0.96	3878
4	2568	0.47	5503
5	700	5.2	136

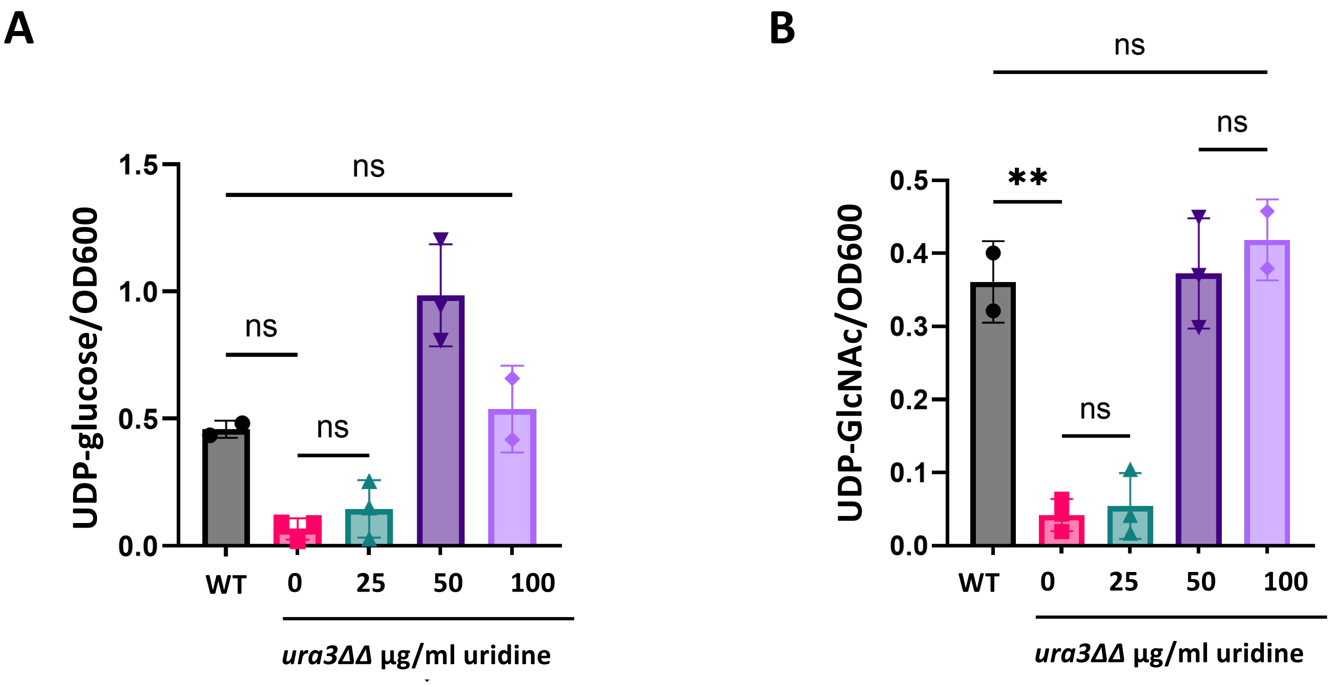
A
UDP-glucose/OD600
1.5
1.0
0.5
0.0
ns
ns
ns
WT
0
25
50
100
ura3ΔΔ µg/ml uridine
B
UDP-GlcNAc/OD600
0.5
0.4
0.3
0.2
0.1
0.0
ns
ns
**
ns
WT
0
25
50
100
ura3ΔΔ µg/ml uridine

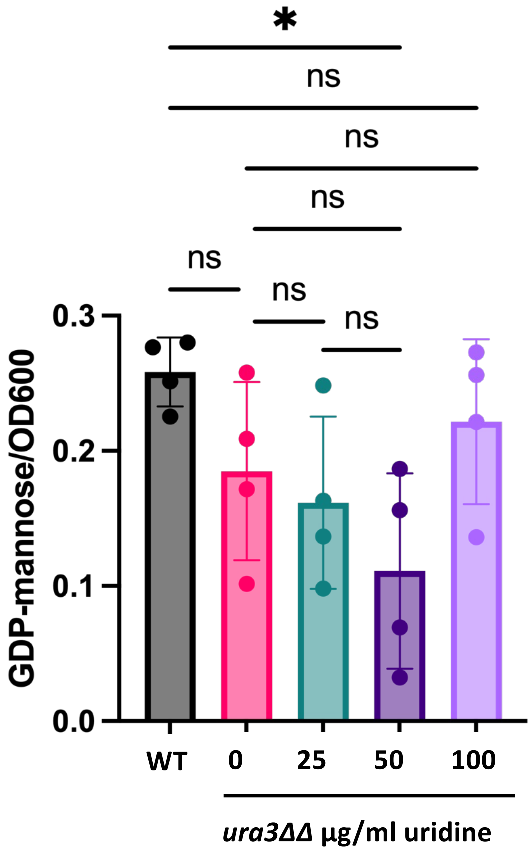
*
ns
ns
ns
ns
ns
ns
GDP-mannose/OD600
0.3
0.2
0.1
0.0
WT
0
25
50
100
ura3ΔΔ µg/ml uridine
