# Supplementary material for: Disruption to de novo uridine biosynthesis alters β-1,3-glucan masking in Candida albicans
Source: mSphere. 2024 Aug 8;9(9):e00287-24. doi: 10.1128/msphere.00287-24 (PMC11423711; doi:10.1128/msphere.00287-24)
Supplement: Legends — for supplemental material. [file msphere.00287-24-s0002.docx]

**Supporting Information**

**S1 Table: Plasmids used in this study.**

**S2 Table: Primers used in this study.**

**S3 Table: *C. albicans* strains used in this study.**

**Supplementary Figure 1: Complementation of CAI-4 with *URA3* restores growth towards wildtype.** (A) SC5314, CAI-4, and CAI4 *ura3ΔΔ::URA3* were grown overnight in YPD and then back diluted and measured for optical density (OD_600_) every 2 hours for the first 10 hours of growth and at the 24 and 48 hour timepoints. Error bars represent the standard deviation. (B) Doubling time was calculated by Malthusian exponential growth from the 4 to 10 hour timepoints. The 95% confidence interval (profile likelihood is also shown). (n=3 biological replicates).

**Supplementary Figure 2: Only supplementation of the *ura3ΔΔ* mutant with uridine can restore β-1,3-glucan masking.** Cells were grown overnight in YPD with 100 µg/mL of uridine, adenosine, cytidine, or guanosine and then stained with an anti-β-1,3-glucan antibody and a phycoerythrin-conjugated secondary antibody for flow cytometry, and median fluorescent intensity of exposed β-1,3-glucan was measured. (n=3 biological replicates with 1 technical replicate for each, **p<0.01, ****p<0.0001, by one-way ANOVA).

**Supplementary Figure 3: The *ura3ΔΔ* mutant exhibits β-1,3-glucan exposure at stationary phase.** Cells were grown overnight in YPD with 0, 25, 50, or 100 µg/mL of uridine then diluted to OD_600_ 0.1 the following morning in fresh YPD with the same concentration of uridine used for overnight growth, then grown for 48 hours. Cells were then stained with an anti-β-1,3-glucan antibody and an Alexa Fluor 488-conjugated secondary antibody for flow cytometry, and median fluorescent intensity of exposed β-1,3-glucan was measured. (n=3 biological replicates with 3 technical replicates for each, *p<0.05, **p<0.01, ****p<0.0001, by one-way ANOVA).

**Supplementary Figure 4: The *ura3ΔΔ* mutant exhibits a unique pattern of β-1,3-glucan exposure at 48 hours.** Wildtype cells were grown overnight, diluted to OD_600_ 0.1 the following morning in fresh YPD then grown for 3 hours and collected for staining. *ura3ΔΔ* cells were grown overnight in YPD and collected for staining or then diluted to OD_600_ 0.1 the following morning in fresh YPD and grown 48 hours for staining. Cells were stained with an anti-β-1,3-glucan antibody and an Alexa Fluor 488-conjugated secondary antibody and imaged using confocal microscopy.

**Supplementary Figure 5: Wildtype and *ura3ΔΔ* cells stained with Concanavalin A show size differences.** Wildtype and *ura3ΔΔ* cells were grown overnight in YPD and measured for median cell size via flow cytometry. (A) Contour plot of wildtype and *ura3ΔΔ* populations showing FSC-A and FSC-W. (B) Median FSC-A of wildtype and *ura3ΔΔ* was measured. (n=9 biological replicates with 1 technical replicate for each, *p<0.05, by Welch’s t-test).

**Supplementary Figure 6: Mkc1 activation is inversely correlated with uridine concentration.** (A-D) Wildtype and *ura3ΔΔ* cells were grown overnight in YPD then diluted to OD_600_ 0.1 the following morning and grown for 48 hours. RNA was extracted for RT-qPCR, and relative transcript abundance of *FKS1* (A), *FKS2* (B), *CHS1* (C), and *CHS3* (D) was measured. (n=3 biological replicates with 3 technical replicates for each, *p=0.0349, **p=0.0035, ***p=0.0005, by Welch’s t-test). (E) Wildtype and *ura3ΔΔ* cells were grown overnight in YPD with 0, 25, 50 or 100 µg/mL uridine, back diluted and grown to mid-log phase (OD_600_=0.8), and protein was extracted for Western Blotting for Mkc1 activation using an anti-p44/42 antibody and an anti-tubulin antibody for loading control. (F) Signal intensities of phospho-Mkc1 in each lane was measured and normalized to tubulin loading control.

**Supplementary Figure 7: UDP-sugar levels are decreased in the *ura3ΔΔ* mutant and are rescued with increasing uridine supplementation.** Wildtype and *ura3ΔΔ* mutant cells were grown overnight in YPD with 0, 25, 50, or 100 μg/mL uridine and analyzed by LC-MS/MS for quantification of (A) UDP-glucose or (B) UDP-*N*-acetylglucosamine. (n=2 or 3 biological replicates, **p<0.01, by one-way ANOVA).

**Supplementary Figure 8: GDP-mannose levels are not altered in the *ura3ΔΔ* mutant.** Wildtype and *ura3ΔΔ* cells were grown overnight in YPD with 0, 25, 50, or 100 μg/mL uridine and analyzed by LC-MS/MS for quantification of GDP-mannose. (n=4 biological replicates, *p<0.05, by one-way ANOVA).
