# Supplementary material for: Disruption to de novo uridine biosynthesis alters β-1,3-glucan masking in Candida albicans
Source: mSphere. 2024 Aug 8;9(9):e00287-24. doi: 10.1128/msphere.00287-24 (PMC11423711; doi:10.1128/msphere.00287-24)
Supplement: Table S1 — Plasmids. [file msphere.00287-24-s0003.docx]

**S1 Table: Plasmids used in this study.**

| Plasmid Name | Genebank Accession | Marker(s) | Reference |
| --- | --- | --- | --- |
| pBSS2 (SAT1 flipper) | ON246334 | Ampicillin, nourseothricin | 60 |
| CaHygB-flipper | ON287367 | Ampicillin, hygromycin | 60 |
