# Supplementary material for: Disruption to de novo uridine biosynthesis alters β-1,3-glucan masking in Candida albicans
Source: mSphere. 2024 Aug 8;9(9):e00287-24. doi: 10.1128/msphere.00287-24 (PMC11423711; doi:10.1128/msphere.00287-24)
Supplement: Table S2 — Primers. [file msphere.00287-24-s0004.docx]

**S2 Table: Primers used in this study.**

| Primer Name | Sequence (5’ to 3’) | Description |
| --- | --- | --- |
| MMO1 | CGGATCTTCAGAACAAAATCC | 5’ UTR forward primer for amplification of *URA3* open reading frame for CAI-4 complementation. |
| MMO2 | GCGGCATTTTCATTATTAGCA | 3’ UTR reverse primer for amplification of *URA3* open reading frame for CAI-4 complementation. |
| MMO5 | GCTAACATCAATAACCCTCTT | Reverse primer in *URA3* open reading frame for complementation screen. |
| crURA3up | AGCAACAACCCCATACACAC | Upstream crRNA for deletion of *URA3*. |
| crURA3down | CCCCTGTCATGATTTCTAGA | Downstream crRNA for deletion of *URA3*. |
| URA3CC9KO-F | TCAATGATGATTTCAACCATTCTTTTAAACATTGATCAATTCCTGGGTTTTCCCAGTCACGACGT | Forward primer for disruption repair template with pBSS2/CaHygB-flipper homology. |
| URA3CC9KO-R | TTTTGAAGATTATAATGATGTTCTTGAAGCGTAAAACATTTGACCGTGTGGAATTGTGAGCGGAT | Reverse primer for disruption repair template with pBSS2/CaHygB-flipper homology. |
| URA3INTF3 | GTTGAAAGTTGCTGTAGTGCCATTG | Forward primer for confirmation of *URA3* deletion. |
| URA3INTR3 | CATCTGCGGCATTTTCATTATTAGC | Reverse primer for confirmation of *URA3* deletion. |
| URA3DETF | GAGAGCAGAAACTCATGCCTCAC | Forward primer for confirmation of *URA3* deletion. |
| URA3DETR | CAGTTCCAGTGCTAACAACTTCATC | Reverse primer for confirmation of *URA3* deletion. |
| FLPINTF | CGCGCGTAATACGACTCACT | Forward primer for confirmation of *URA3* deletion and cassette excision. |
| FLPINTR | CAAGCGCGCAATTAACCCTC | Reverse primer for confirmation of *URA3* deletion and cassette excision. |
| qEPO5 | GATTGGGCTTCATCACCAAC | *ACT1* qPCR forward primer. |
| qEPO6 | GATTGGGCTTCATCACCAAC | *ACT1* qPCR reverse primer. |
| qEPO17 | CCGATGATGTTACTTATCCGG | *CHS3* qPCR forward primer. |
| qEPO18 | CATCTAGCTGCTTGTCGTTCTT | *CHS3* qPCR reverse primer. |
| qEPO149 | CATCGATCCTGACACACCAG | *FKS2* qPCR forward primer. |
| qEPO150 | GACGGTATTTCCCATCAACAAG | *FKS2* qPCR reverse primer. |
| qEPO151 | GAAGCCGCTGAAATCAGATG | *FKS1* qPCR forward primer. |
| qEPO152 | CGAACTTGATTGGCTTCACC | *FKS1* qPCR reverse primer. |
| qEPO153 | GGCTTTCCCACTGTCGACTAG | *CHS1* qPCR forward primer. |
| qEPO154 | GAGGTTGCTGCTCTTGCAG | *CHS1* qPCR reverse primer. |

*Underlined sequences indicate those that bind to pBSS2 and CaHygB-flipper plasmids.
