# Supplementary material for: Disruption to de novo uridine biosynthesis alters β-1,3-glucan masking in Candida albicans
Source: mSphere. 2024 Aug 8;9(9):e00287-24. doi: 10.1128/msphere.00287-24 (PMC11423711; doi:10.1128/msphere.00287-24)
Supplement: Table S3 — Strains. [file msphere.00287-24-s0005.docx]

**S3 Table: Strains used in this study.**

| Strain | Genotype | Parent | Source or Reference |
| --- | --- | --- | --- |
| SC5314 | *URA3/URA3* | n/a |  |
| CAF2-1 | *ura3::imm434/URA3* | SC5314 | 28 |
| CAI-4 | *ura3::imm434/ura3::imm434* | CAF2-1 | 28 |
| DAY286 | *arg4Δ/ARG4 ura3Δ/URA3 his1Δ/his1Δ* | BWP17 | 64 |
| CAI-4 *ura3/ura3::URA3* | *ura3::imm434/ura3::imm434::URA3* | CAI-4 | This study |
| *ura3ΔΔ* | *ura3Δ::FRT+/ura3Δ::FRT+* | SC5314 | This study |
